# Myocardial tagging by Cardiovascular Magnetic Resonance: evolution of techniques--pulse sequences, analysis algorithms, and applications

**DOI:** 10.1186/1532-429X-13-36

**Published:** 2011-07-28

**Authors:** El-Sayed H Ibrahim

**Affiliations:** 1 Department of Radiology, University of Florida - Jacksonville, USA

## Abstract

Cardiovascular magnetic resonance (CMR) tagging has been established as an essential technique for measuring regional myocardial function. It allows quantification of local intramyocardial motion measures, e.g. strain and strain rate. The invention of CMR tagging came in the late eighties, where the technique allowed for the first time for visualizing transmural myocardial movement without having to implant physical markers. This new idea opened the door for a series of developments and improvements that continue up to the present time. Different tagging techniques are currently available that are more extensive, improved, and sophisticated than they were twenty years ago. Each of these techniques has different versions for improved resolution, signal-to-noise ratio (SNR), scan time, anatomical coverage, three-dimensional capability, and image quality. The tagging techniques covered in this article can be broadly divided into two main categories: 1) Basic techniques, which include magnetization saturation, spatial modulation of magnetization (SPAMM), delay alternating with nutations for tailored excitation (DANTE), and complementary SPAMM (CSPAMM); and 2) Advanced techniques, which include harmonic phase (HARP), displacement encoding with stimulated echoes (DENSE), and strain encoding (SENC). Although most of these techniques were developed by separate groups and evolved from different backgrounds, they are in fact closely related to each other, and they can be interpreted from more than one perspective. Some of these techniques even followed parallel paths of developments, as illustrated in the article. As each technique has its own advantages, some efforts have been made to combine different techniques together for improved image quality or composite information acquisition. In this review, different developments in pulse sequences and related image processing techniques are described along with the necessities that led to their invention, which makes this article easy to read and the covered techniques easy to follow. Major studies that applied CMR tagging for studying myocardial mechanics are also summarized. Finally, the current article includes a plethora of ideas and techniques with over 300 references that motivate the reader to think about the future of CMR tagging.

## Introduction

### Heart Disease and Imaging Modalities

Heart disease is the main killer in the western world as it causes considerable morbidity and mortality [1]. More understanding of intrinsic myocardial kinematics could result in improving the interpretation and prediction of changes in different heart conditions. Conventional imaging techniques are limited to assessing the motion of the inner and outer surfaces of the heart wall. Although cardiac function can be evaluated using echocardiography by techniques like speckle tracking, cardiovascular magnetic resonance (CMR) is excelling in terms of tissue contrast, spatial resolution, and signal-to-noise ratio (SNR). Furthermore, echocardiographic imaging makes simplifying geometric assumptions, which are not applicable in distorted heart anatomy. Computerized tomography (CT) is limited by relatively high radiation dose that restricts repeated studies or studies on healthy volunteers. In the past couple of decades, CMR proved to be a valuable and safe tool for cardiovascular imaging [2,3]. Cardiac anatomical, functional, vascular, and metabolic information can be obtained with CMR/MR spectroscopy sequences.

### Global and Regional Cardiac Measures

Regular anatomical images show the inner (endocardium) and outer (epicardium) borders of the heart chambers, which upon segmentation provide valuable measures about the heart global function, e.g. ejection fraction (EF), stroke volume, and myocardial thickness. Although these global measures represent the current standard for evaluating the heart condition, extensive research has shown that regional myocardial functions, e.g. as strain, strain rate, and torsion, allow for early identification of dysfunction, and therefore are becoming extremely important for clinical risk assessment, patient treatment, and therapeutic efficiency. More importantly, many cardiac disorders do not affect the heart wall uniformly, e.g. most ischemic heart diseases affect localized regions of the myocardium. This makes global measures insensitive to alterations in regional performance, and even a normal EF may conceal a significant underlying regional dysfunction. Moreover, since the heart moves through and rotates within any imaging plane during the cardiac cycle, apparent motion of the endocardium and epicardium on tomographic imaging has a complex relationship with intramural myocardial deformation. The lack of intrinsic myocardial markers means that intramural motion components cannot be evaluated by traditional imaging techniques.

### Invasive Techniques for Measuring Regional Myocardial Functions

Originally, measurement of regional myocardial functions required invasive surgical implantation of physical markers within the myocardium and then tracking the implants motion using an imaging modality. Invasive implantation of radiopaque materials [4-7] or ultrasound crystals [8-10] into the heart wall enabled tracking tissue material points within the myocardium and thus measuring local tissue deformation between the tracked markers. However, the implantation process has many limitations: it is an invasive method; it cannot be applied repeatedly; the implants themselves may alter the motion of the tissue in which they are imbedded; and finally, there are only a limited number of material points that can be tracked. Thus, the ability to noninvasively and accurately measure myocardial motion would assist in the diagnosis, prognosis, and management of heart disease.

### CMR Techniques for Measuring Regional Myocardial Functions

The two most widely used CMR techniques for intramyocardial wall motion quantification are myocardial tagging and phase contrast imaging. Although the two concepts appear different, they are closely related to each other, as will be explained later in the review. In fact, different CMR motion measurement techniques are based on magnetization modulating before imaging; and specifically, conventional CMR tagged images represent the special case of acquiring magnitude images after magnetization modulation.

Although it is not the main focus of the current review, a brief description of the main phase contrast methods for measuring myocardial motion is presented here. In 1994, Pelc et al implemented phase contrast CMR for measuring myocardial motion [11]. The method accuracy was evaluated by comparing the resulting measurements with directly visualized motion of CMR signal voids created by implanted markers. Two years later, another method was proposed for tracking myocardial deformation from two-dimensional (2-D) CMR phase contrast velocity maps [12]. The developed method integrated natural spatial constraints of the endocardial and epicardial borders with the phase contrast data to improve measurement accuracy. In 2002, Reese et al presented a three-dimensional (3-D) phase contrast method for measuring strain and strain rate [13]. The developed method resulted in high-quality images in short scan time. Recently, a navigator gated technique was developed for free-breathing, high temporal resolution phase mapping [14]. Several other studies have been conducted that combined phase mapping with different CMR tagging techniques, which will be described later in the article.

### CMR Tagging

The use of CMR tagging for motion tracking has been validated in a variety of phantom studies [15-18], which have compared the CMR measurements to results from other means of motion measurement. CMR tagging has been used in several heart-related and non-heart-related applications as described in [19]. Nevertheless, CMR tagging has been established as an essential technique for measuring regional myocardial function. It allows quantitative measurement of regional intramyocardial motion measures, e.g. strain. The invention of CMR tagging came in the late eighties, when Zerhouni et al [20] introduced a noninvasive technique for creating visible myocardial markers using CMR. The technique allowed for the first time for visualizing transmural myocardial movement without having to implant physical markers. This new idea opened the door for a series of developments and improvements that continue up to the present time. Different tagging techniques are available today that are more extensive, improved, and sophisticated than they were twenty years ago. Current techniques provide high spatial resolution (on the pixel level), high temporal resolution (real-time imaging), and composite imaging capabilities (different information obtained in one acquisition). Famous techniques in use today include spatial modulation of magnetization (SPAMM) [21], delay alternating with nutations for tailored excitation (DANTE) [22], complementary SPAMM (CSPAMM) [23], harmonic phase (HARP) [24], displacement encoding with stimulated echoes (DENSE) [25], and strain encoding (SENC) [26]. Each of these techniques underwent several developments for improving resolution, SNR, scan time, anatomical coverage, 3-D capability, and image quality, which resulted in different versions suitable for various applications.

### Classification of Tagging Techniques

The development of CMR tagging techniques can be broadly divided into two stages. The first stage (basic techniques) started in 1988 with the invention of tagging by magnetization saturation by Zerhouni et al [20], and continued to include SPAMM [21], DANTE [22], and CSPAMM [23] techniques. Other less familiar tagging techniques belong to the 'basic techniques' category, which use tagged rapid gradient-echo magnetization preparation [27], or create ring-shaped tagging for myocardial centerline assessment [28]. The second stage (advanced techniques) started in 1999 by the invention of two of the most widely-used techniques today: HARP [24] and DENSE [25]. This stage includes also SENC [26], which was subsequently developed in 2001. The basic differences between the two stages of CMR tagging development are the concept behind motion decoding and the post-processing criterion used. The techniques in the first stage depend on the creation of a visible pattern of magnetization saturation, usually parallel lines, grid pattern, or radial stripes, on the magnitude reconstructed images. This allow for immediate visual inspection of myocardial contractility without any post-processing. However, exhaustive post-processing is needed to quantify myocardial motion. Different complicated algorithms have been presented for identifying and tracking myocardial tags, which consume long processing time. On the other hand, the techniques in the second stage were rather stemmed from k-space perspective (Fourier Transform (FT) of tagging in the image space), which allows for faster and more automatic analysis of myocardial motion than in the basic techniques. The images resulting from these techniques do not directly show any tagging pattern. However, simple and fast post-processing is needed to yield motion information, which is presented in an intuitive and more appealing way.

### About This Article

Despite the valuable information provided in previous review articles [3,19,29-38], a review article is needed to track the continual technical developments in CMR tagging since the technique was first invented until the current time (Table 1 summarizes evolution of tagging pulse sequences). Current-day techniques are so advanced and complicated that they are hard to comprehend without reviewing the basic blocks on which they were built, and following the incremental developments that led to the present-day techniques, which is the purpose of this article. The current review covers different technical contributions to CMR tagging over more than two decades since Zerhouni's paper was published in 1988. Different developments in pulse sequences and related image processing techniques are described along with the necessities that led to their invention, which makes this article easy to read and the covered techniques easy to follow. Major studies that applied CMR tagging for studying myocardial mechanics are also summarized.

**Table 1:** Evolution of tagging pulse sequences

Technique	Inventor (Ref#)	Year	Advantages	Disadvantages
Magnetization Saturation	Zerhouni (20)	1988	Simple idea; first tagging technique.	Low resolution; long scan time; high SAR.

SPAMM	Axel (21)	1989	Low SAR; available for clinical applications.	Moderate resolution; 2-D only; tag fading.

Cine SPAMM	McVeigh (46)	1992	Cine capability.	Multiple breath-holds.

Localized SPAMM	Chandra (49) Ikonomidou (51)	1996 2002	Tagging confined to region of interest.	Complicated tag preparation.

Variable-density SPAMM	McVeigh (50) Ikonomidou (52)	1998 2003	Sensitive motion estimation.	Long tag preparation time.

Radial tagging	Bolster (54) Bosmans (55)	1990 1990	Suitable for measuring radial strain & heart rotation.	Low resolution for measuring circumferential strain.

Ring tagging	Spiegel (28)	2003	Suitable for measuring circumferential strain.	Low resolution for measuring radial strain.

DANTE	Mosher (22)	1990	High-density pattern of thin tags.	Long tag preparation time.

CSPAMM	Fischer (23)	1993	Improved tagging contrast; no tag fading.	Double scan time as SPAMM.

Slice-following (sf) CSPAMM	Fischer (69)	1994	Tracks tissue through-plane motion.	Lower SNR than CSPAMM.

Single breath-hold sf CSPAMM	Stuber (77)	1999	Fast data acquisition; high temporal resolution.	EPI may cause motion artifacts.

HARP	Osman (24)	1999	Fast tag analysis.	Phase errors; low SNR.

Real-time HARP	Sampath (163)	2003	High temporal resolution.	Complicated setup.

3D-HARP	Pan (166)	2005	3D strain analysis.	Long tag analysis time.

zHARP	Abd-Elmoniem (167)	2005	3D tissue tracking; short scan time.	Complicated data analysis.

fastHARP	Abd-Elmoniem (164)	2007	Short data acquisition time; 25 frames/s.	Complicated setup.

DENSE	Aletras (25)	1999	High spatial resolution; black-blood	Low SNR.

fast-DENSE	Aletras (187)	1999	Single breath-hold.	EPI artifacts.

meta-DENSE	Aletras (181)	2001	higher SNR.	Longer acquisition time.

DENSE with CANSEL	Epstein (184)	2004	higher SNR; less artifacts.	Long scan time.

SENC	Osman (26)	2001	High resolution; simple processing; intuitive view.	Low SNR.

sf-SENC	Fahmy (208)	2006	Through-plane tracking.	Low SNR.

fast-SENC	Pan (209)	2006	Real-time imaging.	Low resolution.

sf-fast-SENC	Ibrahim (210)	2007	Real-time imaging with tissue tracking.	Low resolution & SNR.

C-SENC	Ibrahim (211)	2008	Both strain & viability information in one scan.	No cine capability.

For each tagging technique, the basic pulse sequence is illustrated along with the improved sequences that were developed based on it. The improved sequences are grouped based on the primary improvement goal, i.e., SNR enhancement, scan time reduction, or 3-D extension. Different post-processing algorithms developed for each technique are also covered along with the major applications and research studies that have been conducted based on that technique. As different tagging techniques have distinctive advantages and disadvantages, some efforts have been made to combine different techniques for improved image quality or composite data acquisition. These efforts are also covered in this article. Along this article, similarities and differences between different techniques are pointed out. One advantage of gathering different CMR tagging techniques in one article is that it helps shed the light on their similarities and explore the parallel paths of developments these techniques underwent by different research groups. The current article not only reviews different developments, but also discusses the relationships among them. When looking at the big picture, one observes that although some techniques were separately developed by different investigators whose ideas stemmed from different backgrounds, there exists a common background among these techniques, and each of them can be interpreted from different perspectives. Finally, the current article includes a plethora of ideas and techniques with more than 300 references that motivate the reader to think about the future of CMR tagging.

## Tagging By Magnetization Saturation

**Figure 1:**
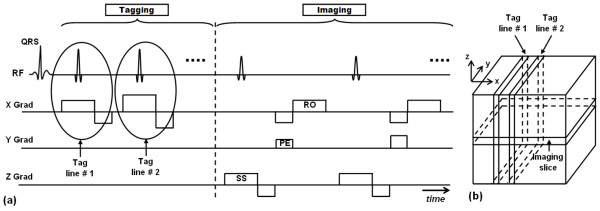
**Tagging by magnetization saturation**. The technique is based on implementing slice-selective magnetization saturated planes orthogonal to the imaging slice. (a) Pulse sequence. Each tagging plane needs a slice-selective RF pulse during the tagging stage. The imaging part follows tagging, and the figure shows conventional Cartesian acquisition (RO = readout, PE = phase encoding, SS = slice selection). (b) Tagging planes and imaging slice.

In 1988, Zerhouni et al [20] introduced the idea of myocardial tissue tagging, which is based on perturbing the magnetization to create visible markers that can be imaged and tracked (Figure 1). The developed pulse sequence consists of two consecutive stages: tagging preparation and imaging. During tagging preparation, slice-selective radiofrequency (RF) pulses are applied perpendicular to the imaging plane to perturb the longitudinal magnetization at specified locations (the intersection of the selected slices and the imaging plane). The rest of the imaging slice are not affected by the tagging pulses and continue to have undisturbed longitudinal magnetization. During imaging (data acquisition), the tagged areas show darker signal intensity than non-tagged tissues due to magnetization saturation they previously experienced. Because magnetization is an intrinsic property of the underlying tissue, the tagged lines, being part of the tissue, follow tissue movement. Thus, the acquired image shows visual evidence of tissue deformation that occurred since the time of tagging pulses application. In the case of myocardium imaging, tagging is typically implemented at end-diastole right after the detection of the R-wave of the electrocardiogram (ECG) signal, and imaging takes place at end-systole, to assess the heart muscle condition at maximum contraction (later, when tagging was combined with cine imaging, it was possible to track the myocardial tagging pattern movement through the entire cardiac cycle).

One point, though, has to be taken into consideration, which is longitudinal relaxation of the tagged magnetization in-between tagging preparation and imaging. Longitudinal relaxation has the effect of restoring the equilibrium condition of the tagged magnetization with exponential rate depending on the tissue longitudinal time constant (T1). Therefore, the longer the time duration between tagging and imaging, the lower the contrast between the tagged and non-tagged tissues in the acquired image.

The tagging technique developed by Zerhouni et al was validated in different studies [16,17]. In [16], the correlation between systolic wall thickening by CMR tagging and by sonomicrometry was examined, and the results showed high agreement between the two techniques. In [17], the relative accuracy of three methods of tag segmentation (manual, automated, and semiautomated) was evaluated along with the methods impact on myocardial strain calculations. The results showed that tag segmentation was extremely reliable for strain measurement.

Although selective excitation was well known long before Zerhouni's paper, the novelty of his work was the use of selective excitation to create visible tissue markers that can noninvasively record intramyocardial motion. However, the need for an RF pulse and an accompanying gradient for creating each tag line rendered the technique impractical for clinical use. For example, to create a stack of ten parallel tag lines that cover the left ventricle (LV), ten slice-selective RF pulses have to be consecutively applied during tagging preparation. This approach has the following limitations: 1) long time is needed for magnetization preparation; 2) the tag lines are not implemented at the same time, which creates intensity variation and non-synchronized tagging deformation in the resulting image; 3) high specific absorption rate (SAR) is deposited into the patient; and 4) low tagging resolution depending on the excited slice profile.

## Spamm

### Original Sequence

**Figure 2:**
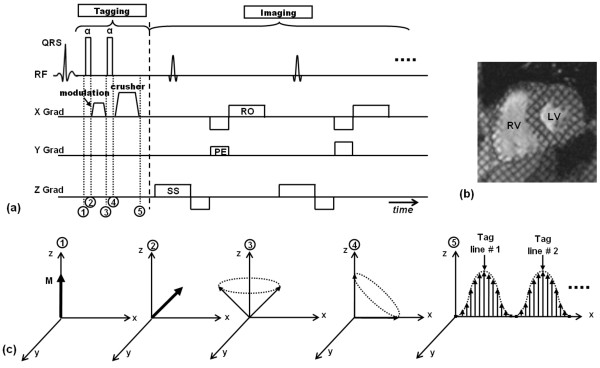
**SPAMM tagging**. (a) SPAMM pulse sequence. The tagging part consists of only two non-selective RF pulses (usually, 90° each), separated by the tagging gradient in the tagging direction, and followed by a large crusher gradient. The imaging part shows conventional Cartesian k-space acquisition (RO = readout, PE = phase encoding, SS = slice selection). This sequence creates parallel tag lines orthogonal to the x-axis. (b) Example of a SPAMM grid-tagged image showing left ventricle (LV) and right ventricle (RV). Note that this grid pattern needs the application an extra tagging stage (in the orthogonal direction) next to the first one before imaging takes place. Note also that the dark myocardium between the tag lines is not completely black due to longitudinal relaxation. (c) Illustration of spins evolution during different time points in the tagging stage, as follows: immediately before tagging application (time point 1), the magnetization (M) is at equilibrium state in the longitudinal direction. Immediately after the application of the first RF pulse (time point 2), the magnetization is tipped into the transverse direction by certain flip angle (45° RF pulses are assumed here for illustration). The tagging gradient then follows, which disperses the spins in the tagging direction (x-direction in this case), such that by the end of the gradient pulse (time point 3), the spins are modulated by incremental phase shifts along the x-axis (the figure shows all vectors emerging from the origin just for simplicity). The second tagging RF pulse tips the resulting modulated magnetization by another 45° into the transverse direction to result in spins modulated as shown at time point (4). A crusher gradient immediately follows to eliminate transverse magnetization components, leaving only the longitudinal parts, which show a sinusoidal pattern along the x-axis with values ranging from 0 to M.

The tagging idea presented by Zerhouni et al led to the invention of a more efficient tagging technique by Axel and Dougherty in the following year, which is still in use until today: SPAMM [21]. The idea behind tags creation with SPAMM (Figure 2) is different from that presented by Zerhouni et al in [20]. SPAMM is based on wrapping the magnetization in a periodic fashion through space by applying only two equal-strength non-selective RF pulses separated by a 'wrapping' gradient. The first RF pulse tips the magnetization into the transverse plane with all spins in phase. A gradient pulse immediately follows along the desired tagging direction. This gradient has the effect of wrapping (modulating) the transverse magnetization in a sinusoidal fashion along the gradient direction through incremental phase shifting of the spins in this direction. It should be noted that the larger the gradient pulse, the higher the tagging frequency. The modulated magnetization is then restored back to the longitudinal position by the second RF pulse. In its simplest form, 90° RF pulses are used to modulate the whole magnetization. Alternatively, RF pulses with less than 90° flip angles could be used for partial modulation, i.e. leaving part of the longitudinal magnetization intact for later use. A large 'spoiler' or 'crusher' gradient follows the second RF pulse to eliminate any remaining transverse magnetization before image acquisition. If grid tagging is required, a second tagging stage (RF pulse/modulating gradient/RF pulse/spoiler gradient) immediately follows the first stage with the modulating gradient orientation orthogonal to that in the first stage. Data acquisition (the imaging stage) occurs later at the desired time point to explore tissue deformation. In its simplest form, the imaging stage consists of a series of slice-selective RF pulses, each followed by phase encoding and readout gradients for k-space filling (Cartesian k-space acquisition). It should be understood that the imaging stage is separate from the tagging stage, and that the tagging pattern experiences deformation based on tissue displacement during the time in-between the two stages. The major achievement of SPAMM is that it made it possible to use myocardial tagging in routine clinical CMR exams. SPAMM imaging was validated in two phantom studies [15,39] and against sonomicrometer measurements in animal models [18], and has been implemented in many research studies [40-44].

### Sequence Developments

In another development by Axel and Dougherty [45], the authors proposed the use of a binomial combination of RF pulses in the tagging stage instead of the special case of two RF pulses, which is referred to as '1-1 SPAMM'. The '1-1' part indicates that the two RF pulses have equal flip angles. In the new development (high-order SPAMM), the tagging part can consist of any number of RF pulses such that their relative flip angles follow a binomial pattern, e.g. 1-2-1, 1-3-3-1, etc. The modulating gradients lie in-between the RF pulses, point in the same direction, and have the same strength as in 1-1 SPAMM. The higher the binomial order, the better (sharper) the tag lines. This development led to improving the tagging pattern quality without compromising SPAMM efficiency. Further SPAMM developments followed by other research groups. In 1992, McVeigh and Atalar developed an imaging sequence for obtaining cardiac cine tagged images in multiple breath-holds [46]. This development was made possible by applying segmented k-space acquisition with multiple views (k-space lines) readout per heart phase. The cine capability allowed for recoding the myocardial contractility pattern through the whole cardiac cycle. In 1994, Reeder et al conducted an interesting study to analyze the tagging contrast and its dependency on the imaging flip angle in breath-hold cine CMR tagging [47]. Recently, the quality of SPAMM tagged images was compared at 3.0 Tesla (T) and 1.5T [48]. The results showed that imaging at 3.0T offers two advantages over 1.5T: 1) SNR is doubled; and 2) myocardial T1 is prolonged, which results in better tagging contrast persistence through the cardiac cycle.

### Localized and Variable-Density SPAMM

Uniform tagging grid that covers the whole image plane may not always be desirable, since motion may be restricted only to specific parts of the image, and also different motion characteristics may call for different tagging grid densities. Localized SPAMM offers an alternative to conventional SPAMM, suitable for applications where the motion to be studied is limited to specific areas of the image. In this case, the tagging grid can be restricted to the area of motion, keeping the rest of the image intact to preserve anatomical information. Some studies had been conducted to localize tagging implementation or to create variable tagging density. In 1996, Chandra and Yang presented sequence variants that may be used for localizing the tagged region within the imaging plane [49]. The developed techniques are useful for optimizing the tagging contrast locally if high tagging density over a small predefined area (within a large field of view) is required. Two years later, McVeigh and Bolster presented a method for producing variable density tagging, capable of producing more sensitive motion estimates than with uniform tag separation [50]. The tag lines separation was customized to match the expected motion of specific regions of the heart wall. With the proposed technique, higher-resolution estimates of both radial thickening and circumferential shortening can be obtained simultaneously. The only limitation of the proposed method is the long time necessary to generate the variable density tagging pattern.

In 2002 and 2003, Ikonomidou and Sergiadis presented two studies about localized SPAMM [51] and variable-density SPAMM [52], respectively. In the first study [51], the authors examined the effect of selective excitation pulses on the SPAMM sequence, and showed that in the case of two identical RF pulses, the phase components are canceled out, and thus preemphasis and refocusing gradients are not needed, allowing for using constant gradients throughout the tagging sequence or choosing nonrefocusable maximum- and minimum-phase RF pulses. In the second study [52], the authors presented a new way of combining 1-1 SPAMM with selective excitation pulses to restrict the tagging grid to regions of interest and produce tagging grid of different density in each region. The method was based on the use of Shinnar-Le Roux selective excitation pulse design algorithm [53], which used the modulus of a finite impulse response (FIR) filter to select the area of the region to be tagged, and its phase to control the grid's density.

### Radial Tagging

It should be noted that although either parallel lines or grid shape are the most used tagging patterns, some investigators suggested radial tag lines for use with cardiac imaging. As early as 1990, Bolster et al developed a tagging method that provided a true polar coordinate system, with both radial and angular dimensions [54]. The proposed method combined the advantages of both radial tagging and SPAMM imaging. The tag lines were placed in the myocardium in a star pattern (when viewed on a short-axis (SAX) slice) such that they intersected in the middle of the LV blood pool. As many as four radial collinear reference points were placed through the myocardial wall at different angles around the LV long axis. The developed sequence provided sufficient radial resolution for studying the transmural dependence of myocardial thickening, and adequate rotational motion sampling for measuring the strain shear components. Six years later, Bosmans et al designed a radial tagging sequence with optimized tagging persistence during the entire cardiac cycle [55]. The authors studied the effects of flow-compensating gradients, excitation flip angles, and flip angles of the saturation pulses on the resulting image quality. In 2001, Peters et al investigated the capability of undersampled projection reconstruction to image myocardial tagging with high spatial and temporal resolutions compared to conventional Fourier transform imaging [56]. The results showed that projection reconstruction could provide high-resolution tagged images with very few projections (scan-time reduction of 1.4 compared to FT) at the expense of some artifacts, which is acceptable as long as the number of projections is large enough to displace the artifacts from the myocardium.

### Tagging Simulations and Analysis

Two studies have been conducted to analyze and simulate the tagging process. In 1997, Crum et al developed an interactive computer program that simulates 2-D CMR tagging [57]. The developed software produces simulated tagged images that can be used for investigating the effect of imaging parameter selection and testing post-processing algorithms. Another theoretical analysis of CMR tagging was presented by Kerwin and Prince in 2000 [58]. In this work, the authors presented a k-space-based approximation that directly related the pulse sequence to longitudinal magnetization. The approximation was particularly useful for design and analysis of tagging sequences. The study demonstrated that the tagging pattern was essentially determined by the autocorrelation of the k-space path function through a simple FT expression. Because many paths often exist with similar or identical autocorrelations, this approach led to a great deal of flexibility in designing tagging pulse sequences.

## Dante

**Figure 3:**
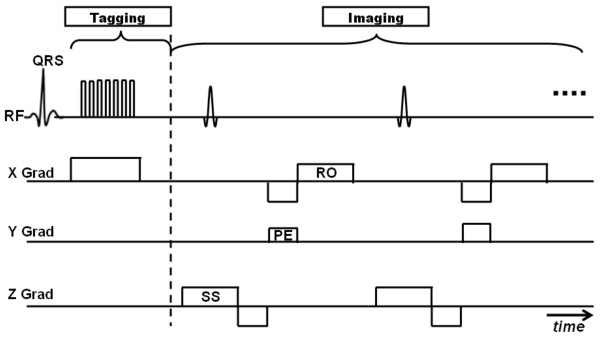
**DANTE pulse sequence**. The tagging stage contains a series of hard (non-selective) RF pulses run simultaneously with accompanying gradient in the tagging direction (1-D tagging is shown here). The imaging stage follows after tagging. The figure shows conventional Cartesian k-space acquisition (RO = readout, PE = phase encoding, SS = slice following).

One year after SPAMM was introduced, Mosher and Smith presented a similar tagging technique, called DANTE, which generates a high-density pattern of thin tags [22]. The technique applies a train of RF pulses in the presence of a continuous gradient to create tag lines in the gradient direction (Figure 3). The flexibility of adjusting the tags spacing and thickness are added advantages of the DANTE sequence. DANTE tagging underwent some developments. In 1995, Tsekos et al developed an improved DANTE technique with B_1_-insensitive adiabatic inversion sequence to generate tags with uniform contrast across the myocardium [59]. Later, Salido et al studied the effects of phase encoding order and segments interpolation on the quality and accuracy of DANTE tags [60]. The results showed that center-out phase order and linear interpolation reconstruction provided the highest tag position accuracy and tag profile quality. In the same year, Wu et al developed a DANTE sequence using sinc-modulated RF pulse train in the presence of constant gradient to improve cardiac tagging [61]. The proposed technique produced rectangular tag profile and offered easier control of the tag width to separation ratio.

## CSPAMM

### Original Sequence

**Figure 4:**
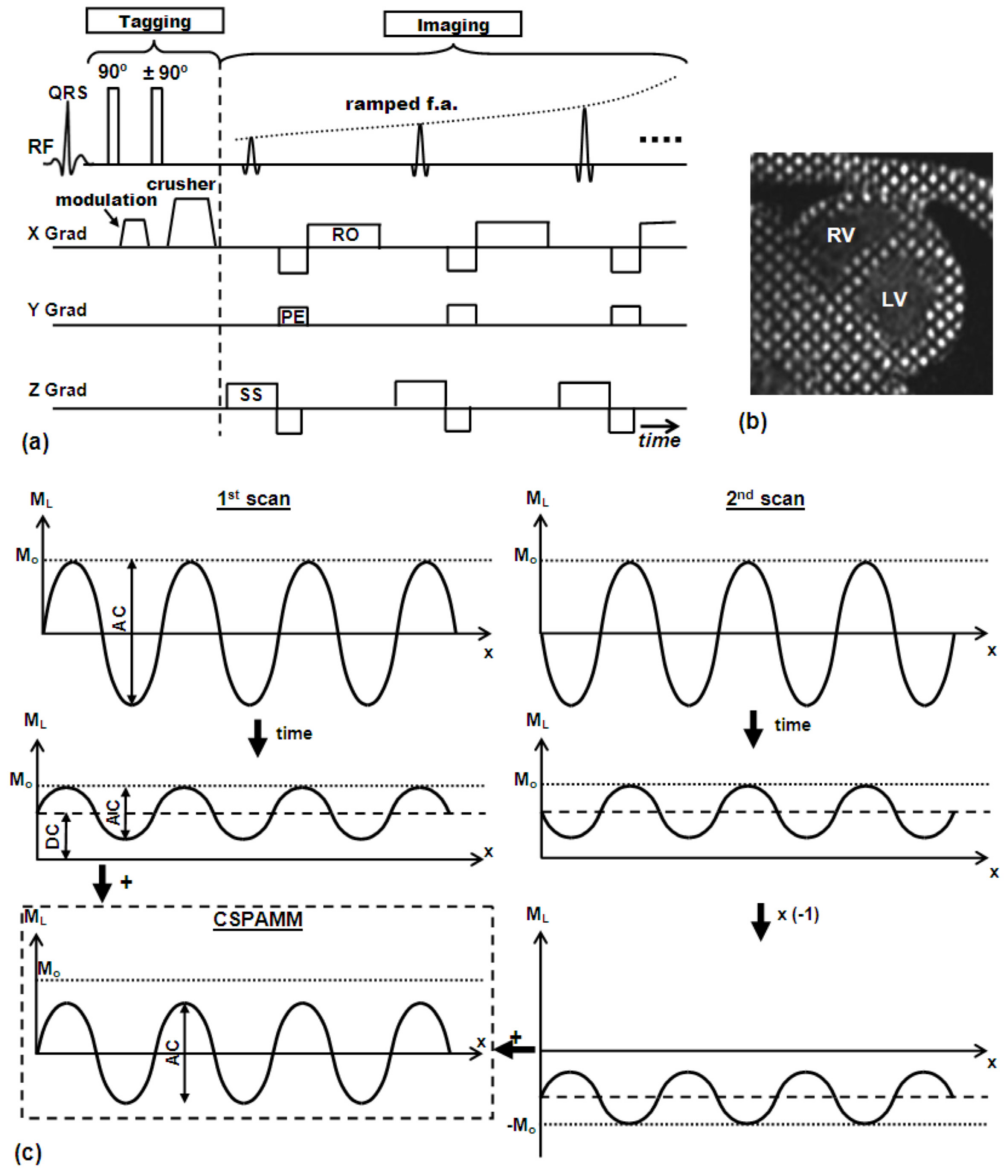
**CSPAMM tagging**. (a) CSPAMM pulse sequence. The sequence runs two SPAMM sequences, with the polarity of the second tagging RF pulse changed in the second SPAMM acquisition. Notice also the ramped flip angles of the imaging RF pulses to compensate for fading tagging. (b) Example of a CSPAMM grid-tagged image. Notice that non-tagged tissues appear black due to the elimination of the offset DC signal. (c) The concept of magnetization subtraction in CSPAMM. Two scans are acquired as shown in the pulse sequence, which results in positive and negative sinusoidal tagging patterns from the first and second scans, respectively. With time, the tagging patterns experience longitudinal relaxation, trying to reach equilibrium (M_0_). The relaxation has two effects on the tagging pattern: the peak-to-peak (AC) magnitude is decreased; and the tagging pattern now has non-zero average (DC) value. However, the DC component is the same in both scans. Thus, at any time point, when the two acquired images are subtracted, the DC component cancels out and the peak-to peak magnitude doubles as shown.

One limitation the tagging techniques presented so far is the fading of the tagging contrast through the cardiac cycle due to longitudinal magnetization relaxation. The loss of tagging contrast towards the end of the cardiac cycle results in unrecognizable tagging pattern, which precludes the analysis of diastolic heart phases. It was not until 1993 when Fischer et al introduced an improved tagging technique (CSPAMM) to resolve this problem [23] (Figure 4). To grasp the idea behind CSPAMM, it is necessary to understand the magnetization evolution with time in a tagging sequence. Immediately after tagging application, the whole magnetization is tagged or modulated (90° RF pulses are assumed) and stored in the longitudinal position. With time, the magnetization experiences longitudinal relaxation, trying to reach the equilibrium state. This has two effects on the stored tagging pattern: 1) introducing a growing non-tagged magnetization offset (we call it here DC component, borrowing the term 'direct current (DC)' from electrical engineering); and 2) reducing the magnitude of the tagged component (the peak-to-peak difference of the sinusoidal tagged magnetization). Thus, during the imaging stage, the excited magnetization has two components: tagged and DC, with the DC overhead impairing the visibility of the (already fading) tagged component. It should be noted that the multiple applications of RF pulses during imaging contributes as well to reducing the tagged magnetization component (each RF pulse consumes part of the tagged magnetization stored in the longitudinal direction). The solution provided by CSPAMM consisted of two parts: 1) eliminating the non-tagged (DC) magnetization; and 2) enhancing the fading tagged magnetization. To eliminate the non-tagged magnetization, two consecutive scans are acquired with exactly the same parameters, except for the polarity of one of the tagging RF pulses. The 90°/90° RF pulses in the first scan modulate the magnetization with a positive sinusoidal pattern, whereas the 90°/-90° RF pulses in the second scan result in a negative sinusoidal pattern. It should be noted that the DC magnetization component is the same in both scans at corresponding time points. Therefore, the overhead DC magnetization can be simply eliminated by subtracting the images in the first scan from the corresponding images (at the same heart phases) in the second scan. This subtraction has also the effect of improving the image SNR by 40% as two acquisitions with independent noise terms are added together. To resolve the second problem of fading tagging contrast, the concept of 'ramped flip angle' was introduced. Basically, during the imaging stage, the flip angles of the RF pulses determine how much magnetization is tipped into the transverse plane for data acquisition. Thus, increasing the flip angles through the cardiac cycle compensates for the fading tagging contrast, because more percentage of the available longitudinal magnetization is used at later heart phases. Fischer introduced a recursive formula for calculating the imaging flip angles based on tissue T1 time constant and the flip angle of the adjacent RF pulse.

CSPAMM tagging was used for analyzing myocardial function in many studies [62-68], and many subsequently developed tagging techniques were based on CSPAMM due to its sharp tag lines and access to late heart phases. The only limitation of CSPAMM is that it doubles the scan time compared to SPAMM.

### Slice-Following CSPAMM

**Figure 5:**
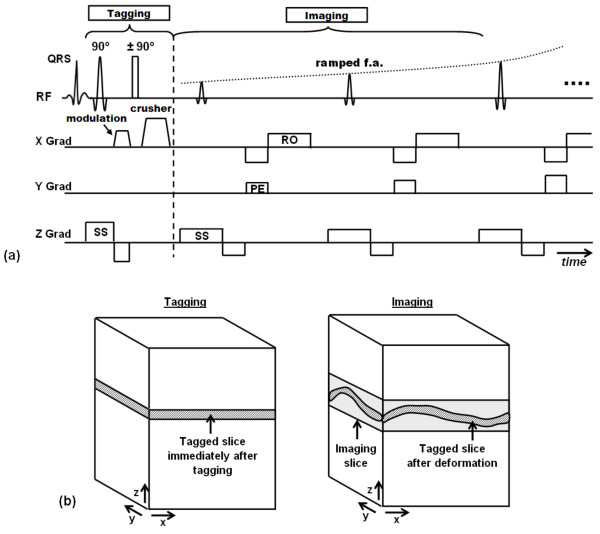
**Slice following**. (a) Pulse sequence. The pulse sequence is similar to CSPAMM, except that one of the tagging RF pulses is replaced by a slice-selective one to create a thin tagged slice. During imaging, a thicker slice is excited. (b) A thin tagged slice is prescribed, which experiences deformation and displacement by the imaging time. A thick imaging slice is selected to accommodate the tagged slice displacement.

Another important contribution by Fischer et al was made in 1994 to resolve the tissue through-plane motion problem [69]. The heart shows a complicated 3-D pattern of contraction through the cardiac cycle. For example, during systole, the myocardium undergoes circumferential and longitudinal shortening, radial thickening, and longitudinal displacement from base to apex. In addition, the heart twists as the base and apex rotate clockwise and counterclockwise, respectively (as seen from the base), which is known as the wringing or torsion heart motion. This means that in 2-D cine imaging of the heart, not the same myocardial tissue is imaged throughout the cardiac cycle [70]. The imaging plane rather shows whatever tissue lies inside it at the time of data acquisition. This could lead to inaccurate assessment of myocardial motion, e.g. apparent myocardial thickening in a basal SAX plane could be in fact due to myocardial basal displacement towards the apex. The slice-following technique [69] was created as an improvement of CSPAMM to resolve the through-plane motion problem (Figure 5). The technique is based on implementing slice-selective tagging instead of the non-selective tagging used in conventional CSPAMM. A thin slice of interest is tagged by switching one (or both) of the tagging RF pulses into a slice-selective pulse, which has the effect of confining the tagging pattern inside the slice of interest. Later, during imaging, a thicker slice, that encompasses the thin tagged slice, is excited. The excited slice should be thick enough to accommodate the thin tagged slice despite its displacement in the through-plane (z-) direction. Because non-tagged magnetization is eliminated in CSPAMM, the only source of signal comes from the initially-tagged slice, regardless of its displacement in the through-plane direction. This ensures that the same myocardial tissue is imaged during the whole cardiac cycle, and that apparent motion illusions are eliminated. The choice of the imaging slice thickness has to be carefully considered to ensure inclusion of the tagged slice throughout the cardiac cycle, and in the meanwhile avoid unnecessary thickness that would only add noise to the image.

It should be mentioned that another idea (slice isolation) was proposed three years earlier for resolving the myocardial through-plane motion problem [71]. In that work, a method was presented for surrounding the heart SAX slice-of-interest with two adjacent parallel saturated slices, and increasing the imaging slice thickness to encompass the slice-of-interest despite its displacement in the long-axis (LAX) direction. The developed technique ensured imaging the same myocardial SAX slice through the cardiac cycle. However, the slice isolation technique is not efficient as the slice-following technique, besides it only applies to SAX slices.

## Data Acquisition Strategies

Different pulse sequences have been developed to address limitations in previous sequences [72]. Gradient echo (GRE) and balanced steady-state with free precession (bSSFP) are the mostly used data acquisition strategies in CMR tagging. Besides, fast data readout techniques, e.g. echo planar imaging (EPI) or spiral, are sometimes implemented for real-time fast imaging.

### GRE

The GRE sequence is one of the most basic CMR imaging sequences [73]. In 1994, Reeder and McVeigh studied the myocardial tagging contrast using rapid GRE segmented k-space cine CMR sequences [47]. The transient behavior of the magnetization signal was measured in a phantom and characterized with Bloch equation simulations for conditions used during breath-hold cardiac tagged CMR. The results showed that the transition to steady state was reproducible after the first heartbeat. Later in 1999, Epstein et al developed a segmented k-space fast gradient-echo pulse sequence with short echo-train readout for high-quality cine imaging of the heart in reduced scan times [74]. Using the developed sequence, cine images of the heart were acquired in as few as 1-5 heart beats and did not display geometric distortion or flow-related artifacts.

### EPI

EPI imaging [75] has also been used for tagging. In 1995, Tang et al [76] introduced the idea of using segmented EPI in CMR tagging to improve myocardial tagging contrast, reduce scan time, and avoid misregistration artifacts from multiple breath-holds. The authors compared the use of multi-shot EPI to segmented spoiled gradient (SPGR) sequences in myocardial CMR tagging. The results showed that EPI could acquire the same amount of tagging data in a shorter time than SPGR. Furthermore, the tagging contrast in EPI was much higher than in SPGR and permitted tag visualization much further into diastole. Four years later, Stuber et al introduced a slice-following CSPAMM sequence in a single breath-hold using segmented EPI imaging [77]. The sequence helped avoid the need for multiple breath-holds with associated motion and misregistration problems. In the same article, Stuber provided a thorough analysis of signal optimization based on the imaging flip angle, heart rate, and number of heart phases. The developed technique allowed for reliable assessment of both cardiac systolic and diastolic phases with high temporal resolution. Another study was conducted in the same year by Reeder et al to improve the tagging sequence by implementing EPI [78]. The authors developed an ultrafast multi-echo hybrid EPI/GRE cardiac tagging sequence with SNR and echo train length optimization. Imaging efficiency was improved by increasing the number of echoes acquired after each RF excitation. The pulse sequence dead periods were minimized using hardware-optimized trapezoid (HOT) gradient pulses. With the developed sequence, significant reductions in total scan time were possible while maintaining good image quality.

Although the hybrid EPI/GRE sequence improved data acquisition efficiency and tagging contrast, off-resonance effects and motion could lead to local phase discontinuities in the raw data when conventional interleaved bottom-up k-space trajectory is used. These discontinuities are particularly problematic for myocardial tagging, where the image energy is not only concentrated near the k-space origin, but also concentrated in multiple spectral peaks centered throughout the k-space. In 2003, Kim et al characterized the tag distortion artifacts in the hybrid sequence due to off-resonance and velocity- induced phase discontinuities [79]. The authors used flyback and gradient moment smoothing methods to reduce these artifacts. Recently, EPI imaging was implemented in CSPAMM by Ryf et al [80]. The developed sequence was optimized for acquisition speed and image quality; thus it combined the advantages of fast read-out and short echo time. It required only two heartbeats and produced sufficiently high image quality, which allowed the sequence to be used in stress studies.

### bSSFP

**Figure 6:**
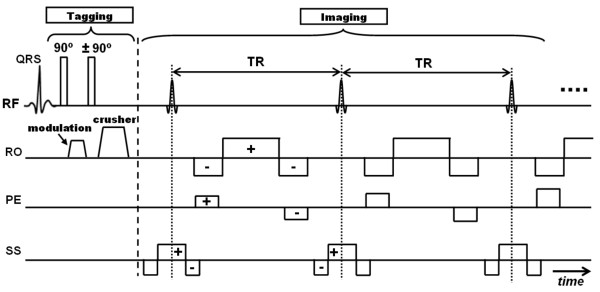
**bSSFP pulse sequence**. The tagging part is similar to regular tagging. However, the imaging part is different, where the gradients are balanced in all three axes. Zero net gradient is achieved during each repetition time (TR) to reduce magnetization dephasing and enhance acquired signal.

The introduction of the bSSFP pulse sequence [81,82] has contributed to CMR tagging improvement. In bSSFP, the remaining transverse magnetization after data readout is re-used during the following repetition times and contributes to subsequent images, in contrast to being eliminated by gradient crushers in SPGR sequence. This is achieved by refocusing (balancing) the gradients in all three axes during each repetition time (TR) (Figure 6). Balanced SSFP proved to be valuable in cardiac imaging due to its high SNR and excellent myocardium-blood contrast [83]. The first implementation of tagging with bSSFP was presented by Herzka et al in 2003 [84]. Implementing the tagging module created a problem as it interrupted the established steady-state condition and resulted in severe ghosting artifacts. Herzka proposed to solve the problem by storing and restoring the magnetization before and after tagging implementation, respectively, using half the flip angle (α/2) technique [85,86]. An optimized flip angle of 40° was used to achieve a compromise between high tagging persistence and high blood-myocardium contrast. The high SNR of bSSFP allowed for improving the tagging contrast and reducing scan time. In a subsequent development by Zwanenburg et al [87], CSPAMM tagging was implemented with bSSFP imaging in a single breath-hold. The linearly increasing startup angles (LISA) technique was implemented for magnetization start-up after tagging preparation. The LISA technique showed more reduction in ghosting artifacts from off-resonance spins (fat) than did the α/2 technique. Zwanenburg opted to use a small imaging flip angle of 20° to optimize the tagging contrast despite the low myocardium-blood contrast, which was enhanced by using the harmonic modulus image to extract myocardium. The tagging bSSFP sequence was two-times faster than the SPGR sequence in addition to the higher tagging contrast.

One problem remained to be solved for the tagging bSSFP sequence: tagging contrast fading through the cardiac cycle. This problem had been previously solved for the SPGR sequence by implementing CSPAMM with the ramped flip angle technique [23]. Unfortunately, the ramped flip angle formula derived for SPGR does not work with bSSFP due to the fundamental differences between the two sequences. Ibrahim et al resolved this issue by deriving a recursive formula for ramped flip angles in bSSFP based on the magnetization trajectory behavior during transition into steady state [88]. The formula was designed to maintain a constant tagging contrast throughout the cardiac cycle. Each flip angle was determined based on the previous flip angle, T1, transverse time constant (T2), and TR times. The final flip angle was numerically optimized to achieve the highest tagging contrast based on heart rate, and to avoid longitudinal magnetization depletion before acquiring data for all heart phases. When compared to SPGR with ramped flip angles, the ramped flip angle bSSFP sequence provided double the tagging contrast during the same scan time [88]. In 2007, Johnson et al conducted a study to assess the diagnostic value of myocardial tagging with bSSFP, and concluded that bSSFP was superior to the gradient echo sequence [89]. The improved tagging contrast and tag persistence with bSSFP facilitated post-processing and enabled diastolic function analysis.

Another contribution to tagging with bSSFP was made by Derbyshire et al, who developed a phase-sensitive myocardial tagging sequence without extending the scan time [90]. The proposed sequence resolved the problem of rectified inverted tags in magnitude- reconstructed images when 90°/90° SPAMM is implemented. This problem leads to false tagging nulls when the signal crosses zero, which reduces the apparent tag spacing in half. After using the phase associated with the tag peaks, the tagging contrast improved, which allowed for using fully-automated algorithms for tracking the tag lines.

### Other Data Acquisition Strategies

Besides GRE and EPI sequences, other data acquisition strategies have been implemented with tagging. In [56], the authors implemented tagging with radial acquisition, and they were able to achieve high resolution tags with 40% reduction in scan time, compared to Cartesian sampling. Spiral acquisition is another data sampling strategy, which has many advantages including efficient signal sampling with small number of excitations, reduced sensitivity to flow artifacts due to the self-refocusing gradients, short echo time (TE), and isotropic spatial resolution. Due to its nature, high-frequency data (k-space periphery) is sufficiently acquired, which maintains the high spatial resolution needed for visualizing tagging details. In 2004, Ryf et al implemented CSPAMM tagging with interleaved spiral imaging, which allowed for improving spatial and temporal resolutions [91]. The resulting images demonstrated enhanced tagging contrast throughout the cardiac cycle.

Another technique for improving the tagging resolution was proposed by Stuber et al, who developed a modified CSPAMM technique [92]. The developed technique allowed for shifting the tagging grid to any position in the imaged slice. The tagging density was doubled by acquiring two tagged images with the tagging grid of the second image shifted by half cycle (half the distance of tag separation) with respect to the first one, and then adding the two images. With this technique, it was possible to avoid the Nyquist theorem limitation, even in the presence of large tissue contraction.

## Tagging Analysis

Basic tagging sequences, especially SPAMM, are usually implemented in clinical applications when information is needed about local myocardial contractility, e.g. in the presence of infarction or ischemia. Usually, three sets of SAX images (covering basal, mid-ventricular, and apical locations) and one set of four-chamber (4CH) cine grid-tagged images are acquired for evaluating LV myocardial contractility. Although advanced sequences provide more accurate results and need simpler post-processing than basic sequences, they are mostly used in research studies. However, some exceptions exist, e.g. measuring myocardial strain in the RV or obtaining different information in the same scan. The acquired tagged images are transferred to a computer system for processing and analysis. The basic idea behind all tagging analysis techniques is tracking adjacent tag intersection points and measuring relative increases or decreases of their in-between distances from time frame to another to calculate strain. Various strain components are measured throughout the cardiac cycle: circumferential and radial strains are measured from SAX images, while longitudinal strain is measured from 4CH images. The resulting strain curves show the myocardial contractility pattern during different heart phases. Important cardiovascular parameters can be extracted from the resulting curves, e.g. peak strain and its timing, which help in evaluating the heart condition. Furthermore, strain rate could be obtained by differentiating myocardial strain with respect to time, which gives useful information about the degree of heart dysfunction, e.g. evaluating myocardial relaxation rate in diastolic dysfunction. Occasionally, myocardial twist angle may be obtained by measuring circumferential strain difference between parallel SAX slices. This parameter sheds light on the heart efficiency in ejecting blood during systole. Different CMR software packages, e.g. FindTags [93] and Diagnosoft (Diagnosoft, Inc., Palo Alto, CA, USA), are available for analyzing tagged images and calculating different contractility parameters.

Different post-processing techniques have been developed for extracting and tracking myocardial tags in cine tagged images [94]. The amount of post-processing performed to analyze the tagged images ranges from simple visual inspection of tag deformation to exhaustive calculations of strain components. Many efforts have been made to facilitate myocardial motion quantification from tagged images. Semiautomatic methods for tracking tag deformation include active contour models, optical flow techniques, and template matching methods. Table 2 shows a summary of different tagging analysis techniques. Active contour methods depend on applying spline curves (snakes) that are semi-automatically fitted to the tag lines through the combination of internal, external, and user-interactive forces. Optical flow techniques depend on tracking the tagging grid intersections, which have signal intensity minima, from one frame to another. Finally, template matching methods estimate tissue deformation by cross-correlating a pre-defined tagging pattern with the tagged images. The work by Kraitchman et al in [95] represents an example of combining matching template and active contour techniques for semiautomatically tracking myocardial motion in CMR tagged images.

**Table 2:** Tagging analysis techniques

Method	Characteristics	Advantages	Disadvantages	Ref #
Active contour	Uses spline curves that are fitted to the tag lines using multiple constraints.	Intuitive approach; parametric continuity; local control of the curve shape.	Long processing time; sensitive to weights of different constraint forces.	93, 96, 97, 101, 102.

Optical flow	Tracks tag lines intersections based on tagging contrast.	Possibility for automatic processing; reduced processing time.	Sensitive to image quality, especially tagging contrast.	103-105, 107-110.

Template matching	Cross-correlates a pre-defined tagging pattern with the resulting images.	Reduced processing time.	Pre-defined assumptions must be met.	95.

Sinusoidal analysis	Data are analyzed into different frequency components.	Decreased sensitivity to noise; high accuracy.	Complicated data analysis.	111-113.

Volumetric modeling	Analyzes a stack of parallel tagged images.	3-D tagging analysis; more automatic processing.	Long processing time.	134-139.

Finite-element modeling	Creates model tags, which define the tag lines in the images.	3-D tagging analysis; reduced processing time.	Measurements are not directly related to clinical understanding.	140-142.

Statistical modeling	Uses statistical methods for estimating tag lines deformation.	3-D capability; more intuitive and understandable parameters.	Predefined assumptions; complicated processing.	141, 143.

3-D active contour modeling	Uses 3-D spline curves that are fitted to tag lines from a set of parallel images.	3-D capability; high resolution; parametric continuity.	Long processing time.	144-148.

### Active Contour Methods

As early as 1994, Guttman et al proposed a template-matching method to detect tags and an active contour method to extract myocardial contours [96]. Based on this approach, a software package called FINDTAGS [93] had been created for extracting and tracking tag lines; however, it required several hours to process one dataset. Also, in 1994, Kumar et al and Goldgof et al presented an approach for automatic tracking of SPAMM tags [97]. In this approach, snakes were used for spatio-temporal tracking of the SPAMM grid-tagged pattern. Correspondences between the grid points in consecutive frames were used with a thin-plate spline model to establish mapping from one image to the next (non-rigid registration)[97]. Recently, Montillo et al presented different techniques for segmenting and processing SPAMM tagged images [98-100].

Base (B)-spline curves were also used to represent tag lines. B-splines can describe more than one-dimensional tag displacements and characterize deformations of the image plane, volume-of-interest, or space-time continuum. The use of B-splines for representing tag lines has several advantages, including compact representation, parametric continuity, and local control of the shape (only the locations of few control points need to be optimized in order to determine the location of a complete tag line). In 1998, Amini et al used coupled B-spline grids to track tag deformations [101]. In his work, new techniques were described for efficient reconstruction of dense displacements from SPAMM grids. The intersection points of the SPAMM grids were treated as standard landmarks and were forced to align. The developed method resulted in accurate measurements of in-plane tissue deformations as well as between any two frames in a sequence of tagged images in a reasonable time.

In 1998 also, Stuber et al presented an evaluation tool for the visualization and quantification of local heart wall motion from tagged CMR images [63]. The first processing step was tag line detection using active contour models. The second step involved definition of sensitive motion parameters, their visualization, and the relevance of these parameters to heart wall motion. The developed visualization tools allowed for detailed investigation of locally and temporally resolved heart wall dynamics. A couple of years later, Ozturk and McVeigh [102] presented a method for describing the heart motion using a four-dimensional (4-D) tensor product of B-splines on CMR tagged images. The proposed method had the following advantages: it used information from all available tag data; it had a compact parametric representation; the calculated deformations were continuous both in time and space; it did not use a chamber-specific coordinate system; and it was relatively fast (10 minutes of processing time per image).

### Optical Flow Methods

Optical flow techniques have been used in several studies for tracking myocardial tagging. In 1992, Prince and McVeigh presented an optical flow method for reconstructing motion from a sequence of CMR tagged images [103]. The developed method (variable brightness optical flow (VBOF)) was used for motion estimation with compensation for tagging pattern decay. The magnetic resonance imaging equation of the tagged images was used to provide an estimate of the material time derivative, which was used for optical flow calculations. The developed VBOF method was markedly superior to standard optical flow methods on tagged images with decaying tagging contrast. A few years later, Dougherty et al developed an optical-flow method for rapid estimation of myocardial displacement from CMR tagged images [104]. The developed method (registration and change visualization (RCV)) used a hierarchical estimation technique for computing the flow field that describes the warping of an image at certain heart phase to the next image. The proposed method did not rely on prior knowledge of the image content and overcame the requirement of constant pixel intensity in standard optical flow methods. Another contribution by Prince et al came in 2000 [105], where the authors developed a fast, fully automated optical flow method for tracking CMR tagging pattern by exploiting the Fourier content of the tagged images. The developed method worked by extracting various sub-band images from the tagged cardiac data, and then formulating multiple optical flow constraints for each sub-band. The resulting system of equations was then solved by least squares pseudo-inversion. The proposed method was validated on simulated and real tagged data.

Besides FT, Gabor filter [106] was used for tagging analysis. In 2006, Qian et al developed a method for automatically extracting the tag lines in tagged CMR images and tracking their displacement during the heart cycle using a tunable 3-D Gabor filter bank [107]. The Gabor filter bank was designed based on the geometric characteristics of the tag lines, and its tunable parameters were used to adapt to the myocardium deformation. The whole image dataset was convolved with each Gabor filter in the filter bank. A set of deformable meshes was imposed onto the extracted tag lines and tracked over time; and dynamic estimation of the filter parameters and the mesh internal smoothness were used to help the tracking. Another optical flow-based method was proposed by Denney et al in 2003 [108]. The method used a maximum-likelihood/maximum a posteriori technique for tag detection and strain calculation without applying user-defined contours. The developed technique reduced the occurrence of false tag detections, and significantly reduced the processing time.

Recently, Herrezuelo et al presented a method for motion estimation of tagged cardiac CMR sequences based on variational optical flow techniques [109]. The phase of the tagged images was used to perform accurate and robust tracking by incorporating the motion estimates of control points with high phase stability into the approach. Another method was also recently proposed by Florack and van Assen for myocardial tagging analysis based on a multiscale algorithm that exploits local scale selection to obtain estimates of the velocity gradient tensor field [110]. Time evolution of the deformation tensor was governed by a first-order ordinary differential equation, which was completely determined by the velocity gradient tensor field. The authors solved the set of ordinary differential equations analytically and presented results from healthy volunteers and patients. The proposed method required only off-the-shelf algorithms and was readily applicable to planar or volumetric tagging CMR data sampled on arbitrary coordinate grids.

### Sinusoidal Analysis

Other methods have been proposed for tag tracking based on sinusoidal analysis. In 1996, Zhang et al presented a method for automatically tracking SPAMM tag lines on gated cardiac images [111]. The developed method used Fourier based spatial frequency and phase information to separately track horizontal and vertical tag lines. The use of global information from the frequency spectrum of the entire set of tag lines resulted in a robust algorithm with decreased sensitivity to noise. A few years later, Clarysse et al developed a method for tracking spatio-temporal myocardial displacement using a cosine series model fitted to the entire tagged dataset [112]. Various spatio-temporal parameters were computed, which provided a set of motion features, e.g. trajectories of material points or velocities of deformations over time. The proposed method, combined with a specific visualization tool, provided an innovative way for noninvasively analyzing myocardial contractile function.

Recently, Arts et al developed another method for extracting motion from CMR tagged images based on sinusoidal approximation [113]. In the developed method, the environment of each pixel in the tagged image was modeled as part of a sine wave with local frequency and amplitude. The image intensity in the environment of each pixel was modeled as a moving sine wavefront, and displacement was estimated at subpixel accuracy. The proposed method resulted in displacement estimates with high accuracy and reduced noise.

## 3-D Tagging

Different efforts have been made to extend the basic tagging technique into 3-D or conduct 3-D myocardial motion analysis from multiple 2-D tagged images. Various studies have been conducted using 3-D CMR tagging, as will be explained later in the 'Applications' Section [43,114-125]. A couple of articles were published in 2000 and 2001 about different methods of 3-D reconstruction and modeling of the heart motion [126,127]. In 2000 also, Moore et al conducted an interesting study about 3-D systolic strain patterns in normal LV using orthogonal sets of tagged images [128,129]. The study presented an important database of systolic 3-D strain measurements in normal LV.

### 3-D Tagging Sequences

**Figure 7:**
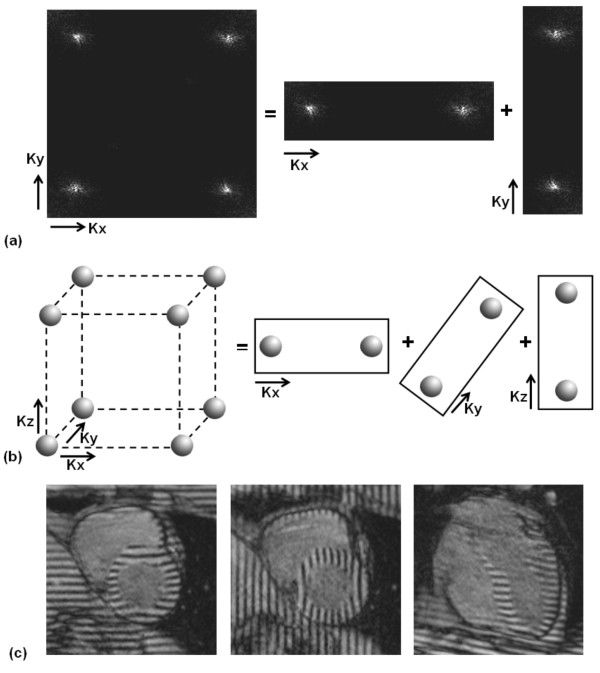
**Tagging by consecutive application of 1-D tagging in orthogonal axes**. (a) 2-D case. Instead of applying tagging in two orthogonal axes in the same scan (to create a grid tagged pattern), two 1-D tagging scans are conducted consecutively, so that only a small portion of k-space that includes the signal peaks is acquired each time, instead of acquiring the whole k-space in the first case. (b) Extension to 3-D case. Instead of applying tagging in three orthogonal directions in the same scan, three consecutive 1-D tagging scans are conducted consecutively to significantly save data acquisition time. (c) The three orthogonal 1-D tagged images. Short-axis slices are tagged in both horizontal and vertical directions in two separate acquisitions, and four-chamber images are tagged in the horizontal direction. The displacement information from all three sets of images are combined together to obtain 3-D displacement information.

Extending conventional tagging techniques into 3-D is a straightforward process with the major limitation of prolonged scan time. Few attempts have been conducted to achieve this goal. In 1995, Perman et al developed a technique for 3-D tracking of myocardial motion in a selected slice by combining in-plane DANTE tagging with through-plane motion detection using phase contrast [130]. The developed protocol allowed for determining point-specific myocardial strain values in vivo. 3-D versions of CSPAMM have also been presented in [131,132]. In 2002, Ryf et al combined CSPAMM with 3-D modulation of the magnetization and 3-D EPI imaging [131]. Later in 2008, Rutz et al provided an accelerated technique for whole-heart 3-D motion tracking using CSPAMM [132]. However, instead of implementing 3-D modulation as in [131], the authors implemented consecutive one-dimensional (1-D) modulations in three orthogonal directions (Figure 7). This allowed for partial k-space acquisition without compromising spatial resolution, as only the regions of k-space that contained tagging information were acquired after each tagging application. The developed technique reduced scan time, and allowed for assessment of 3-D motion information with whole heart coverage in three short breath-holds.

Similar to the work by Perman et al [130], Sampath et al have recently combined SPAMM with phase-contrast imaging, however, this time the developed sequence was used to provide simultaneous measurements of LV longitudinal strain and chamber blood velocity for any given LV LAX slice in a single, short breath-held acquisition [133]. In the proposed technique, the images corresponding to odd-numbered cardiac phases were standard 1-1 SPAMM tagged images, while the even cardiac phase images included a velocity-encoded phase term in addition to motion-related tagging modulation. Since the blood velocity and myocardial strain data were acquired simultaneously, any transient physiological events, e.g. induced stress, were manifested in a correlated fashion on both datasets.

### 3-D Tagging Analysis

Different methods have been developed for analyzing 3-D myocardial motion (Table 2 shows a summary of different analysis techniques). Each method has its advantages and disadvantages with respect to robustness, 3-D interaction, computational complexity, and clinical interpretation. In comparison to other forms of tag representation, the use of active contours showed several advantages, including immediate generation of tag surfaces, subpixel accuracy of tag line localization, parametric continuity, and the ability to determine the location of a complete tag surface by assigning the location of few control points. Nevertheless, there is always a tradeoff between the temporal and spatial resolutions each method offers, which makes the method choice depending on the application at hand and on the investigator needs.

#### Volumetric modeling

3-D analysis of myocardial motion requires the presence of volumetric tagged data. Usually, stacks of orthogonal SAX and LAX slices are combined to allow for tracking tag lines deformation in 3-D. The accuracy of 3-D motion measurements have been examined in orthogonal tagged images of a rotating phantom as well as in the heart wall [134]. The results showed that through-plane motion (in the z-direction) can be measured as accurate as that of in-plane motion. Later, Kuijer et al proposed the acquisition of multiple SAX and LAX views for 3-D myocardial motion analysis using global interpolation and smoothing [135]. The proposed method showed to be robust in segments with poor or incomplete tagging data, and was able to detect small regions of contraction.

One way to measure 3-D myocardial deformation is by combining separate displacement fields fitted to orthogonal one-dimensional tagged images (Figure 7). In [136], O'Dell et al used three sets of orthogonal tagged images. The first and second sets consisted of SAX slices with horizontal and vertical tags, respectively, while the third set consisted of 4CH slices with horizontal tags. 3-D motion information was calculated from the 3-D displacement field fitted to the orthogonal tagged images. Alternatively, the stack of planar tagged images can be processed together for more unified displacement analysis.

Denney et al presented a series of developments for myocardial 3-D motion analysis from CMR tagged images [108,137-139]. In 1997, Denney and McVeigh presented a method for myocardial motion analysis through model-free reconstruction of 3-D myocardial strain from planar tagged images [137]. The myocardial volume was decomposed into a dense mesh of points using a discrete algorithm, and a high-resolution 3-D displacement field was reconstructed using finite difference analysis. Strain was then calculated by numerically differentiating the reconstructed displacement field. A few years later, Denney et al presented another improvement by developing a method for unsupervised reconstruction of 3-D myocardial strain from parallel tagged images [108]. The method consisted of an automated tag tracker followed by an automatic estimation of the endocardial and epicardial contours. Strain was then reconstructed using the previously developed discrete model-free algorithm [137]. In 2004 and 2005, Deng and Denney [138,139] presented other methods for 3-D myocardial motion analysis. Instead of tracking the tag lines independently in each slice before reconstructing myocardial deformation, the proposed methods fitted a 3-D myocardial deformation model directly to the tagged images, which ensured that the tag positions identified in the images were consistent from slice to slice.

#### Finite element modeling

**Figure 8:**
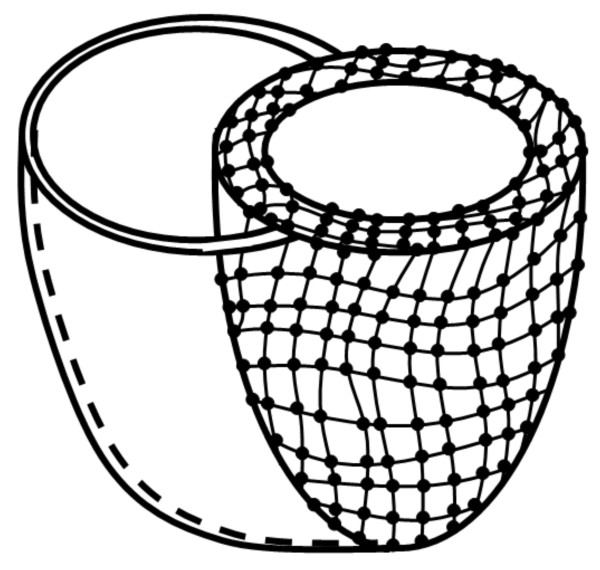
**Heart 3-D modeling**. The spline lines (or planes) are fitted to the tag planes, and their intersections create marker points that are used to build a 3-D finite-element model of the heart. The model is used for displacement calculations.

Finite element modeling is a typical choice for volumetric motion analysis, since it provides strain analysis throughout the ventricular wall (Figure 8). In [140], Young developed a method for direct 3-D myocardial tracking from tagged images without prior identification of ventricular boundaries or tag line locations. The method utilized a finite element model to describe the heart shape and motion. Model tags were created as material surfaces, which defined the location of the tag lines. An objective function, of the difference between the model tags and the image stripes, was derived and minimized to allow the model to deform to the tag lines in the images. The proposed method reduced the processing time significantly compared to methods that separately track the tag lines in each slice. A few years later, Hu et al presented another method that used a finite element model for calculating myocardial 3-D motion from tagged images [141].

In addition to the numerous studies that analyzed the LV, two studies were conducted to analyze the right ventricle (RV) motion using finite element models. In 1996, Young et al described a method for reconstructing the RV free wall 3-D motion using CMR tagging [142]. In the developed method, tag intersection points were tracked through systole in both SAX and LAX images. A finite element model of the midwall surface of the RV free wall was constructed to fit the midwall shape at end diastole. The model was then deformed to each subsequent frame by fitting the tag displacements and midwall contour locations. A few years later, Haber et al described a method for reconstructing the 3-D motion of the RV by fitting a deformable model to tag and contour data extracted from multiview CMR tagged images [121]. The deformable model was a biventricular finite element mesh built directly from segmented contours. The proposed approach accommodated the geometrically complex RV by using the entire length of the tags, localized degrees of freedom, and finite elements for geometric modeling.

Despite of its advantages, one limitation of finite element representation is that it does not directly lend itself to an understanding of the underlying kinematics in a clinically useful way. In order to understand the complex relationship between the resulting measurements and other motion parameters, it is desirable to express the motion in terms of meaningful physical parameters that offer sufficient accuracy.

#### Statistical modeling

Statistical methods have also been used for estimating 3-D myocardial motion. In 1993, Hu et al developed a statistical model for estimating in vivo strain and stress of both cardiac ventricles by using accurate displacements reconstructed from CMR tagged images and a deformable model [141]. Based on the motion data reconstructed from CMR tagging and previously measured in vitro myocardial material properties, active force was estimated, from which in vivo material properties were assessed. By iterating these calculations, maximum likelihood estimations of the active force and the material properties were obtained. The finite element method was then used to calculate strain and stress. Compared to traditional methods that use strain energy functions, the proposed method gave more intuitive and understandable parameters. Another statistical method was proposed by Chen and Amini in [143], where the authors presented a maximum a posteriori framework for detecting tag lines using Markov random field defined on the lattice generated by 3-D and 4-D (3-D + time) uniform sampling of B-spline models. First-order smoothness constraints were used as *a priori *on the displacement field and myocardial contours. The developed method had many advantages including user interaction and 4-D model fitting capabilities.

#### 3-D active contour modeling

Active contours have also been used for tracking 3-D myocardial deformations from parallel CMR tagged images. In 1998, Kerwin and Prince presented a method for generating accurate motion estimates over a sparse set of material points using parallel CMR tagged images [144]. Each tracked point was located at the intersection of three tag surfaces, each of which was estimated using a spline. The tagging intersections were determined using an iterative projection algorithm with a convergence criterion. The proposed method allowed for fast processing time because motion estimates were derived from sparse sets of material points instead of producing dense motion estimates. In the following year, Huang et al proposed a 4-D B-spline model for spatio-temporal tracking of myocardial deformations from tagged images [145]. A system was built, which takes as an input the tag lines extracted from SAX and LAX image sequences, and fits time-varying B-spline models to the data. An advantage of the proposed method is that tag surface reconstruction, 3-D material point localization, and displacement assessment are all performed in one step. Similar to the method developed in [144], Wang et al proposed a method for fast LV motion estimation using subspace approximation techniques [146]. In the developed technique, multidimensional interpolation was implemented to reconstruct dense displacement fields from the sparse measurements. Displacement estimation was formulated as a variational problem and the solution was projected on spline subspaces. Efficient numerical methods were derived based on B-spline properties, which allowed the algorithm to be implemented in fast time.

Amini et al presented several studies about using active contours for 3-D analysis of myocardial deformations [147,148]. In [147], the authors described efficient methods for encoding, visualization, and tracking of 3-D myocardial taglines in the heart from two sets of orthogonal tagged MR views using 3-D B-splines. A couple of years later, Tustison et al presented a technique for employing 4-D B-splines for determination of tagline intersections and myocardial strains [148]. The developed 4-D B-spline model was specified by a 4-D grid of control points to interpolate the tag information across space and across all continuous time points. Data from each time frame was represented by a 3-D B-spline model specified at certain time point. The developed model was capable of producing comprehensive myocardial strain calculations by reconstructing 3-D displacement fields based on available tag information.

## HARP

### Original Sequence

**Figure 9:**
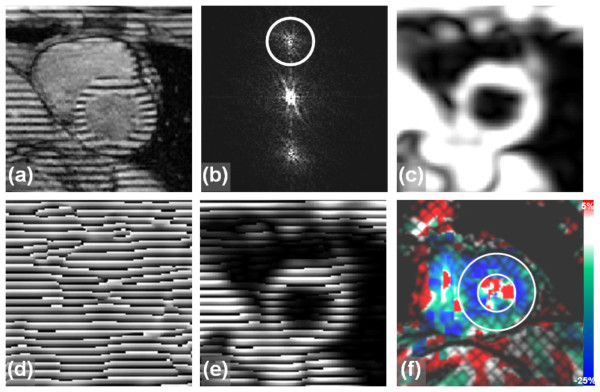
**HARP tagging**. (a) Original SPAMM tagged image. (b) K-space of the tagged image. HARP applies a spatial bandpass filter to extract only the first harmonic peak. (c) Magnitude HARP image is created by Fourier transforming the modified k-space and obtaining the magnitude data. (d) HARP phase image is created by Fourier transforming the modified k-space and obtaining the phase data. (e) Multiplying the HARP magnitude and phase images results in an image with modulation pattern very similar to the original tagged image. However, the pattern here represents the wrapped tissue phase. Myocardium displacement tracking can be conducted by simply tracking the tissue phase from frame to frame. (f) An example of a grid-tagged image analyzed with HARP, which shows myocardial circumferential strain.

In 1999, Osman et al introduced HARP as a method for processing SPAMM tagged images and extracting myocardial motion measurements [24,149,150] (Figure 9). HARP is based on the fact that tag implementation modulates the underlying image with spectral peaks in the k-space at multiples of the tagging frequency. The idea in HARP is to isolate the signal peak at the first harmonic frequency, which contains the tagging information. After applying FT, both harmonic magnitude and phase images are obtained. The harmonic phase image shows intensity gradients interrupted by sharp transitions resulting from phase wrappings every 2π, while the magnitude image shows a blurred anatomical mask of the myocardium. When multiplied together, the phase and magnitude images result in a new image (the HARP image), which is very similar to the original tagged image. As the harmonic phase is a material property of the tagged tissue, the intensity pattern in the HARP image closely follows the tagging pattern deformation in the tagged image. Myocardial motion estimation can be then achieved by simply tracking the phase of the point of interest in a small region of interest (ROI) around it after phase wrapping correction. This technique resulted in automatic and fast analysis of tagged images. Strain measurements by HARP have been validated against measurements from conventional tag analysis techniques [151]. Furthermore, tag analysis by HARP showed high degrees of robustness and reproducibility as shown in the results from the Multi-Ethnic Study of Atherosclerosis (MESA) study [152], and has been used in several research studies [152-154].

### Sequence Developments

In his work to improve HARP, Kuijer et al showed that more accurate results and fewer artifacts resulted when HARP was applied on CSPAMM images instead of conventional SPAMM [155]. The key point is that the central peak in k-space is effectively suppressed in CSPAMM, which allows for more flexible choice of the cut-off frequency of the band-pass filter used to isolate the tagging spectral peak. In this case, higher filter bandwidth would not include data from the central peak, the matter that usually generates striping artifacts in the resulting images. Similar to the work by Kuijer et al, another contribution to improve HARP image quality was made by Ryf et al [156]. However, instead of working on the central peak, the authors proposed to combine the positive and negative harmonic peaks to correct for phase errors and improve SNR before conducting the HARP analysis. The investigators showed that the resulting images were free from phase distortion artifacts, and the reproducibility of strain measurements was improved.

As any 2-D imaging technique, HARP suffers from tissue through-plane motion, which results in that some tissues do not show during the whole cardiac cycle due to the heart longitudinal motion and its conical shape. In [157], Tecelao et al presented an extended HARP technique to measure strain values from myocardial regions that appear only during the systolic phase of the cardiac cycle. The authors designed a mathematical model of myocardial deformation using Hilbert Transform to quantify the accuracy of the developed technique in strain measurements. Recently, Bilgen developed a technique for improving tag line detection using special phase manipulation [158]. A special combination of wrapped phases was applied to the HARP image to create an image with unique intensity pattern that can be exploited for automatically detecting tag intersections. The developed technique allowed for strain measurements at higher spatial resolution and multi-scale.

### HARP: Tracking Improvement

In addition to the HARP developments that stemmed from k-space perspective, other developments were created to improve motion tracking using HARP. In 2004, Osman et al developed a method for regenerating tagged images using HARP [159]. The synthesized tag lines showed crisp profile than the original sinusoidal pattern. In 2005, Khalifa et al proposed to combine HARP with active contours to improve the analysis process, especially at low SNR levels where tracking errors are expected [160]. Recently, some contributions have been made that followed similar reasoning lines. In [161], Liu and Prince presented a refinement of the HARP technique that included a spatial continuity condition to avoid motion estimation errors, which may result from large deformations, through-plane motion, or at tissue boundaries. An optimal tracking order was sought from a certain seed point to each point in the image based on the shortest path principle, which encouraged the search to stay within the same tissue region as the seed point. The proposed method considerably minimized motion tracking errors, and showed to be robust and fast.

### HARP: SNR Improvement

An important source of errors in HARP imaging is due to additional phase values present in the reference time frame. In [162], Li and Yu developed a HARP-based method for restoring undeformed taglines at the reference frame for accurate motion quantification using finite element analysis. The developed technique utilized the fact that the location of the off-center spectral peaks is dependent on spatial modulation frequency. Therefore, a single point of the spectral peak maximum could be used to fully recover the undeformed taglines. The study showed that strain calculations without correcting for early myocardial deformation in the reference frame lead to significant underestimation of ventricular contraction

### HARP: Reduced Scan Time

Efforts have been made to reduce the scan time associated with HARP analysis. Sampath et al presented a real-time version of HARP using multi-coil data acquisition, EPI imaging, and fast data processing on a computer unit next to the scanner [163]. A complete set (different frames throughout the cardiac cycle) of 1-D SPAMM tagged images were acquired during each heart beat with temporal resolution of 40 ms using single-shot EPI free-breathing imaging. Alternating horizontal and vertical tagged images were acquired on consecutive heart beats to construct grid-tagged images. Corresponding images from consecutive heart beats were combined and processed to result in grid-tagged strain images at a rate of four images per second. In a similar work, Abd-Elmoniem et al presented the FastHARP technique, which produced real-time strain images at a rate of 25 frames/s [164]. As in Sampath's work, horizontal and vertical tagging were applied alternatively in consecutive heart beats, and combined together to update strain calculations every heart beat. However, instead of acquiring the whole k-space and extracting the spectral peak of interest using a band-pass filter as in conventional HARP, the authors proposed to acquire only a small part of k-space around the spectral peak of interest, the matter that significantly reduced data acquisition time. To further accelerate the display rate, image formation was accomplished using custom-designed chirp inverse FT with a priori designed lookup tables to reconstruct only a small ROI around the heart.

### 3-D HARP

**Figure 10:**
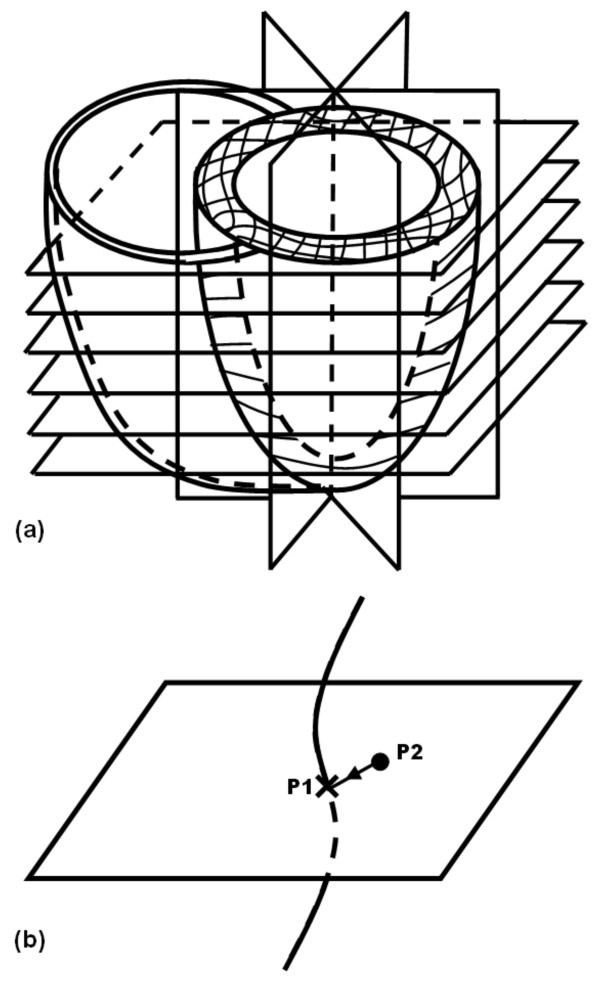
**3D-HARP**. (a) Two series of tagged images are acquired: 1) a stack of parallel short-axis grid tagged images to obtain in-plane x-y displacements; and 2) a radial set of long-axis line-tagged images to obtain through-plane (in the z-direction) displacements. (b) The HARP concept is used to track myocardium. The intersection of tag line with the image plane (point P1) is linked to the nearest point on the next frame with the same characteristic phase (point P2).

The efforts to improve HARP were not restricted to reducing scan or processing times only, but extended to allow for 3-D strain analysis. In 2002, Ryf et al [131] and Haber et al [165] proposed 3-D myocardial motion analysis techniques based on extensions of 2-D HARP. Ryf et al [131] applied the extended HARP technique to 3-D CSPAMM imaging, while Haber et al [165] derived a 3-D finite element model for estimating myocardial 3-D motion from multiple 2-D tagged images. In 2005, the 3D-HARP technique was proposed by Pan et al as a straightforward extension to HARP for fast and semiautomatic tracking of myocardial 3-D motion [166] (Figure 10). In 3D-HARP, a parallel set of SAX grid-tagged images and a radial set of LAX images with horizontal tags were acquired to construct a 3-D mesh around the LV. Motion tracking was automatically conducted using a 3-D version of the phase-invariance concept used in HARP. As a further step to minimize error accumulation during the transition from timeframe to another, the phase of each material point was checked at each timeframe against its initial value. The 3D-HARP method compared well to other validated techniques, and provided LV myocardial 3-D motion analysis in about 10 minutes with very little human interaction.

**Figure 11:**
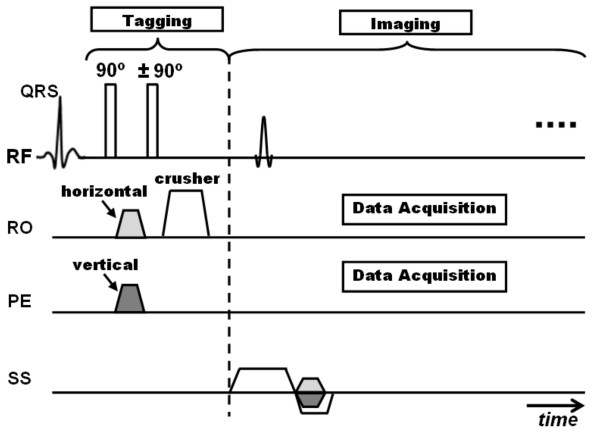
**zHARP pulse sequence**. The pulse sequence is derived from CSPAMM. Two CSPAMM images are acquired with horizontal and vertical lines to obtain in-plane strains. Through-plane strain is obtained by adding positive (light gray) and negative (dark gray) z-gradients after the slice selection pulse of the horizontal and vertical tagged images, respectively.

Another important extension of HARP for 3-D motion analysis was presented by Abd-Elmoniem et al [167,168]. The technique was called zHARP, and it allowed for myocardial 3-D strain quantification from a single image plane using tagging and phase encoding for deciphering in-plane and through-plane information, respectively (Figure 11). The technique was based on slice-following CSPAMM without increasing scan time. The first set of CSPAMM images (two SPAMM scans with horizontal tags) was modified by adding a small z-encoding gradient to the slice-selection refocusing pulse. An opposite polarity z-encoding gradient was added to the second set of CSPAMM images (two SPAMM scans with vertical tags) to encode the through-plane motion information. The horizontal and vertical tag lines provided in-plane motion information, while the added z-encoding gradients resolved motion in the z-direction. As CSPAMM imaging was used, susceptibility and field-inhomogeneity related phase artifacts were eliminated after image subtraction. In-plane and through-plane motion components were extracted by applying HARP to positive and negative harmonic peaks (total of four harmonic peaks) and solving the resulting set of equations. The advantage of zHARP is that it provided 3-D motion information about the slice of interest in a short time (four short breath-holds) without the need to acquire orthogonal sets of tagged images. Later, Abd-Elmoniem et al extended zHARP into a multi-slice technique for rapid 3-D strain tensor quantification [169]. To achieve this goal, a stack of SAX zHARP images were acquired and processed to derive a stacked array of 3-D strain tensors without the need to image multiple orientations or use numerical interpolation. 3-D strain mapping of the whole heart was calculated in less than 20 s.

It should be noted that the idea of combining tagging and phase- or velocity-encoding, for simultaneous acquisition of in-plane and through-plane myocardial motion information, has been previously provided by Perman [130] and Kuijer [170], respectively. Recently, two studies have been conducted for improving 3-D motion analysis with HARP [171,172]. The first study was conducted by Venkatesh et al, who developed a new method for phase unwrapping in HARP for 3-D strain measurement of the entire LV [171]. Phase residues in the HARP images were used for automatically detecting phase inconsistencies in the wrapped phase images. With the proposed technique, tagged images covering multiple slices were acquired every breath-hold, which allowed for entire LV strain analysis in short time and with high accuracy. The second study was presented by Chuang et al for improving strain measurement sensitivity with less susceptibility to noise [172]. The authors developed a method that combined CMR tagging, automated material point tracking, and finite element models for calculating 3-D strain distributions in the mouse LV using HARP. The developed method showed high degrees of sensitivity and accuracy.

## DENSE

### Original Sequence

**Figure 12:**
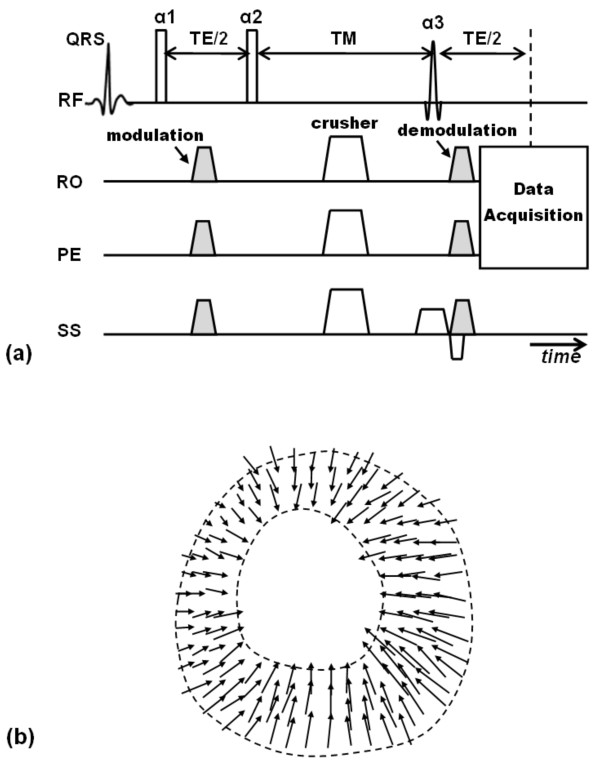
**DENSE**. (a) DENSE pulse sequence is derived from STEAM sequence. Displacement encoding (modulation) and decoding (demodulation) gradients are added after the first and third RF pulses, respectively. Both gradients have equal magnitudes, and they are applied in the direction where displacement information to be obtained. During the mixing period in-between modulation and demodulation, large crusher gradients are applied to dephase any transverse magnetization, such that the remaining magnetization is stored in the longitudinal direction, where it experiences only longitudinal relaxation. The time between the first and second RF pulses is equal to the time between the third RF pulse and data acquisition (= echo time (TE)/2). TM = mixing time; RF = radiofrequency; RO = readout; PE = phase encoding; SS = slice selection. (b) Example of DENSE image of the left ventricle, where representative vectors are drawn at each pixel, with the vector magnitude and direction represent the displacement value and orientation, respectively.

Similar to HARP, DENSE was introduced in 1999 by Aletras et al to measure tissue motion from phase data [25] (Figure 12). However, DENSE evolved as a Stimulated Echo Acquisition Mode (STEAM) pulse sequence [173] with displacement encoding, in contrast to HARP, which was directly associated with tagging. In fact, although HARP and DENSE were separately developed and presented as evolving from different backgrounds, they have more similarities than differences, and they represent two sides of the same coin. In their interesting article, Kuijer et al evaluated and compared the two techniques as two approaches to phase-based strain analysis [174].

As a STEAM pulse sequence, DENSE consists of three stages: modulation, mixing, and demodulation. During the modulation part of the sequence, longitudinal magnetization is tipped into the transverse plane, encoded with modulation gradient, and then stored back into the longitudinal direction until imaging takes place. Magnetization storage in the longitudinal direction allows for less relaxation and more tagging persistence. Tissue displacement occurring during the mixing period is recorded in the imaging stage. During imaging, the modulated magnetization is excited (tipped into the transverse plane) and decoded using gradient with the same magnitude as the modulation gradient. Stationary spins are perfectly rewound and have zero net phase. However, spins that moved during the mixing period in the gradient direction accumulate phase due to their different spatial positions during modulation and demodulation. By acquiring another DENSE image with different modulation value and subtracting the two phase images, displacement information in the gradient direction can be decoded. For 3-D motion analysis, four DENSE images are required (one reference and three images encoded in orthogonal directions).

DENSE reveals motion information on the pixel level by displaying a small vector at each pixel location. The vector orientation and length represent the motion direction and magnitude, respectively. One advantage of DENSE is that it is a black-blood sequence due to the disturbance of the modulated pattern in blood during the mixing period, which facilitates myocardium border identification. From k-space perspective, DENSE positions the displacement-encoded echo at the k-space origin, while other extraneous echoes are shifted or suppressed with the goal of sampling the uncorrupted displacement-encoded echo with high spatial resolution. Numerical simulation and in vivo experiments have been conducted to validate DENSE accuracy [175]. The results indicated that strain measurements by DENSE are highly reproducible and quantitatively equivalent to measurements from conventional myocardial tagging sequences. The sequence has been used in several research studies [176-178].

### DENSE: Effects of Imaging Parameter

Recently, two articles have been published that investigated the influence of different imaging parameters on DENSE performance. In DENSE, the phase of the stimulated echo is influenced by off-resonance and B_0 _inhomogeneity in addition to the encoded displacement. If phase shifts from sources other than the encoded displacement are not accounted for, they lead to errors in the measured tissue motion. Therefore, instead of the absolute phase, multiple measurements are usually acquired to obtain the desired phase shifts that represent tissue motion. In [179], Haraldsson et al studied the influence of the unencoded free induction decay (FID) and off-resonance effects on DENSE acquisitions. The study showed that the accuracy of DENSE CMR can be severely affected by the unencoded FID and off-resonance effects. However, these errors can be significantly reduced by using an encoded reference, e.g. an encoded complementary strategy. Phase alteration gives the FID and stimulated echo different phases, which makes any influence of the FID easier to identify than in normal situations where the phase of these signal components are correlative. The study recommends that DENSE users should evaluate the FID influence and off-resonance effect on their DENSE protocols.

In [180], Sigfridsson et al studied the mutual SNR influences of flip angle strategies, field strength, and spatial variation of receiver coil sensitivity on DENSE imaging. The results showed that these different aspects are associated with different tradeoffs. The effect of field strength and receiver coil sensitivity influences the SNR with the same order of magnitude, whereas flip angle strategy can have a larger effect on SNR. Thus, careful choice of the imaging hardware and adaptation of the acquisition protocol is important for achieving sufficient SNR in DENSE CMR. The proposed study can be potentially used as a guideline for parameters optimization in specific DENSE applications.

### DENSE: Different Echoes

**Figure 13:**
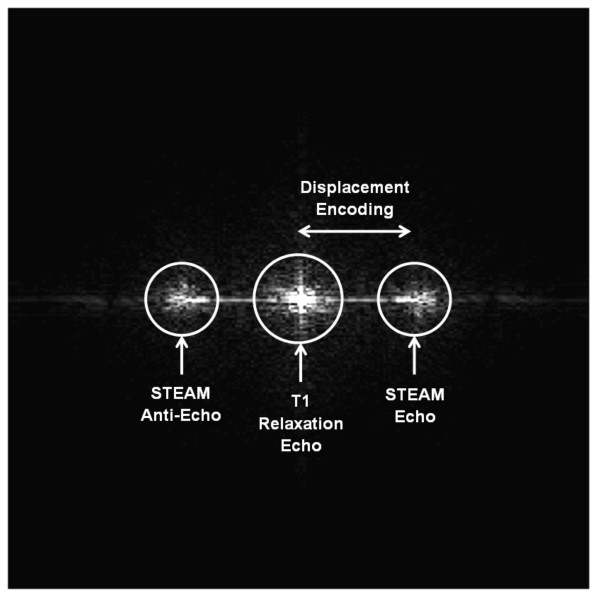
**DENSE k-space**. Three echoes are generated in DENSE: stimulated echo, stimulated anti-echo, and T1 relaxation echo. Only the stimulated echo contains the displacement information of interest, while other echoes are to be suppressed. The bigger the displacement encoding frequency, the larger the separation between the stimulated echo and the T1 relaxation echo, which results in displacement-encoded image not corrupted by other signals. The T1 relaxation echo can be also suppressed by inversion recovery or phase cycling (as in CSPAMM). The anti-echo may be added to the stimulated echo after phase correction to improve SNR.

Similar to HARP, DENSE [25] also underwent a number of improvements for enhancing SNR, reducing scan time, and extending to 3-D imaging. Methods for enhancing SNR generally deal with the resulting signal echoes. In DENSE, three echoes generally result: the stimulated (displacement-encoded) echo, the complex conjugate of the stimulated echo (anti-echo), and an echo due to T1 relaxation (non-modulated DC echo) (Figure 13). Only the phase of the displacement-encoded echo is directly related to tissue displacement. Thus, it is usually the case that only the stimulated echo is acquired, since otherwise the additional echoes will cause errors in displacement measurement. Some of the proposed methods add the echoes in a correlative way to enhance the recorded signal, while others suppress unwanted echoes using various techniques to avoid artifacts.

The anti-echo is often suppressed using large displacement-encoding gradient such that the spatial frequency of the anti- echo is greater than the frequency range detected during data acquisition. Alternatively, this echo can be suppressed using phase cycling techniques, e.g. CSPAMM; however, this requires extra acquisitions. In theory, one could also suppress the longitudinal relaxation echo by using even larger displacement-encoding gradients such that the spatial frequency of the T1 relaxation echo is greater than the frequency range detected during data acquisition. However, in practice, this requires displacement-encoding gradients that are very large, which causes dephasing and signal loss of the desired stimulated echo during imaging of a contracting heart. More practical techniques for suppressing the T1 relaxation echo include inversion recovery, CSPAMM, and other phase cycling techniques. Inversion recovery suppresses the echo only at a certain time point, and thus is not applicable to cine DENSE. Subtracting data from complementary acquisitions, as in CSPAMM, suppresses the T1 relaxation echo independently of time and longitudinal relaxation; however, this approach requires two acquisitions and can experience incomplete suppression with variable heart rate. Recent advances in phase cycling are capable of isolating the stimulated echo and the artifact-generating echoes separately; however, the number of acquisitions should be at least equal to the number of echoes, which results in long acquisition times.

### DENSE: SNR Improvement by Echo Combination

Different techniques have been proposed for enhancing DENSE SNR through combining echoes in a coherent way. In 2001, Aletras and Wen presented the mixed echo train acquisition (meta)-DENSE technique [181]. meta-DENSE is based on DENSE with mixed echo acquisition, which results in improved data acquisition speed and spatial resolution. Modified fast spin echo readout was implemented in meta-DENSE to ensure that any contributions to the acquired signal from local stimulated echoes add in a coherent manner. Inversion recovery was applied to eliminate unencoded signals, and gradient waveforms were balanced between consecutive 180° pulses to ensure successful artifact removal. It should be noted that meta-DENSE allowed for longer readout periods than conventional cine DENSE because the T2* transverse relaxation is replaced by T2 relaxation in meta-DENSE. In a further development to suppress the anti-echo signal in DENSE, Aletras and Arai combined meta-DENSE with RF phase cycling in [182]. In the modified sequence, both the real and imaginary components of the magnetization were acquired in successive acquisitions and combined to construct the whole position-encoded complex signal. The improvement in image quality obtained by both suppressing the T1 relaxation echo using inversion recovery and suppressing the anti-echo signal using RF phase cycling resulted in strain maps with significantly reduced artifacts.

Echo combination is another idea for improving DENSE SNR that was proposed by Kim et al in [183]. Basically, DENSE suffers from low SNR because it is a STEAM pulse sequence, which acquires only half the data containing the modulated information. The idea behind DENSE echo combination is to extract a pair of sub-sampled modulated images from a complex tagged image, and combine them during image reconstruction. Because the two acquired images have uncorrelated noise, their addition leads to SNR enhancement. The pair of sub-sampled images was extracted from a CSPAMM tagged image by splitting the raw data matrix into positive and negative parts. The two images were separately reconstructed using inverse FT and then added together. The resulting averaged image was used to calculate myocardial displacement and strain as in conventional DENSE. With the proposed echo combination technique, net SNR improvement ranged from 14% to 34% throughout the cardiac cycle. It should be noted that the DENSE echo combination idea is similar to the peak combination idea previously described for HARP [156], and that both techniques were developed, again separately, at the same year 2004.

### DENSE: SNR Improvement by Echo Suppression

A number of techniques have been proposed for enhancing DENSE SNR by suppressing unwanted echoes. In 2004, Epstein and Gilson presented DENSE with cosine and sine modulation to eliminate (CANSEL) the T1-relaxation and complex conjugate echoes using both cosine and sine magnetization modulations [184]. The developed technique suppressed the artifact-generating echoes without constraining the displacement-encoding frequency or direction. The use of CANSEL technique lead to improved SNR and the ability to accurately measure through-plane as well as in-plane tissue displacements. The disadvantage of the proposed technique is the long scan time needed for multiple acquisitions.

A couple of years later, Zhong et al modified the cine DENSE sequence to include dephasing gradients and perform CSPAMM [185]. Through-plane gradients were added to the sequence to selectively dephase artifact-generating echoes without significantly affecting the stimulated echo. The proposed technique for suppressing the artifact-generating echoes was independent of T1 and displacement-encoding frequency, and did not require additional acquisitions. It should be noted that the induced through-plane spatial frequency must be limited when imaging the heart; otherwise, a greater signal loss may be experienced due to cardiac deformation. Another limitation of the proposed technique is that through-plane dephasing cannot be used when displacement encoding is applied in the through-plane direction or when volumetric 3-D DENSE is performed.

Recently, a general balanced multipoint method was presented for improving SNR in n-dimensional DENSE [186]. The developed method provided reduced phase noise variance for a given displacement encoding frequency, and eliminated the direction bias in phase noise. However, one limitation of the proposed method is that more phase wrapping occurs compared to the simple multipoint method. The balanced multipoint method was compared to the simple multipoint method, and the results showed that the proposed method decreased phase noise covariances by over 99% and reduced the direction bias to almost zero.

### DENSE: Reduced Scan Time

In addition to the efforts described for improving DNESE SNR, other efforts were made to reduce DENSE scan time. In 1999, Aletras et al presented a single-breath-hold DENSE (fast-DENSE) technique for measuring myocardial strain in a single slice [187]. The sequence included a multishot segmented k-space EPI sampling scheme, and was tested on human subject. Data processing required minimal user intervention, and the resulting measurements had sufficient resolution to reveal transmural strain variation across the LV wall. In 2004, Kim et al presented a single breath-hold high spatial-resolution 2-D cine DENSE sequence [188]. Three cine datasets were acquired in each encoding direction: a phase reference dataset and two complementary displacement-encoded sets. The complementary datasets served to suppress T1 relaxation artifacts. Displacement encoding was implemented in two orthogonal directions by swapping the readout and phase encoding directions. Fast EPI imaging was applied, which allowed for acquiring each cine dataset in three heartbeats.

Parallel imaging techniques have been also combined with DENSE for accelerating data acquisition. In [189], Aletras et al described a method for combining DENSE with SENSE acceleration [190] to reduce total breath-hold time. A single non-encoded low resolution B_1 _map was acquired for reconstructing all three complex images (two orthogonal directions plus reference), which allowed for more acceleration when compared to potentially collecting a separate map for each of the three DENSE images. The developed method allowed for obtaining high-quality strain maps within an appropriate scan time for clinical use. More importantly, the implemented SENSE acceleration did not change the strain noise level when compared to unaccelerated scans. Later, an integrated technique for improving DENSE SNR and data acquisition efficiency was presented by Kim and Kellman [191]. The developed technique combined the use of echo-combination image reconstruction, bSSFP imaging, sensitivity encoding (SENSE) parallel imaging, and 3.0T field strength. The implementation of parallel imaging allowed for accelerating cine DENSE acquisition rate by a factor of two, and resulted in a total acquisition time of 12 heartbeats. The use of bSSFP, echo combination, and 3.0T field strength improved SNR and compensated for losses associated with parallel readout.

Recently, Zhong and Yu developed a 2-D multiphase DENSE method for direct quantification of Lagrangian strain, and implemented their method for imaging the mouse heart under dobutamine stimulation [192]. The developed method allowed for direct cardiac strain quantification at both baseline and high workload with high spatial (0.56 mm) and temporal (< 10 ms) resolutions.

### 3-D DENSE

Different developments have been achieved to allow for 3-D motion tracking using DENSE. In the first development, Gilson et al developed a novel pulse sequence based on DENSE to achieve multislice coverage, high spatial resolution, and 3-D displacement encoding [125]. The developed method assessed 3-D myocardial mechanics in less than one hour of scan time. Data analysis was straightforward, with manual intervention required only to segment the LV myocardium. Later, in 2008, Spottiswoode et al presented a method for 3-D tracking of myocardial motion using slice-following cine DENSE imaging [193]. This development allowed for obtaining true 3-D frame-to-frame motion trajectories of material points initially lying inside the imaging plane, and the authors were able to quantify myocardial through-plane tissue rotation.

Another DENSE development was presented by Zhong et al for 3-D myocardial strain imaging using navigator-echo volumetric cine DENSE with spiral acquisition [194]. The navigator echo technique allowed for free-breathing imaging, and spiral 3-D acquisition (stack of 2-D spirals) allowed for TE minimization, SNR improvement, and data sampling efficiency. Balanced displacement encodings were implemented for SNR improvement. Fat suppression and three-point phase cycling were applied to reduce artifacts from unwanted signals. One limitation of the proposed pulse sequence was its long scan time, which could limit the sequence applicability. Recently, Sigfridsson et al presented a modified DENSE sequence for single breath-hold (18 heartbeats) multi-slice strain imaging [195]. The key point in the modified sequence was position-encoding the whole heart during the modulation stage, followed by multiple position decodings of different slices. A total of three slices were acquired using three EPI excitations each. Displacement encodings were applied in three oblique directions with interleaved phase cycling to subtract background phase errors.

### DENSE: Composite Information Acquisition

The DENSE sequence has been modified to acquire more information in addition to motion measurement. In 2004, Gilson et al developed a modified DENSE sequence for simultaneous acquisition of myocardial displacement-encoding and contrast-enhancement information from the same raw data by acquiring two complementary DENSE images [196]. Myocardial displacement measurements were obtained from the phase-reconstructed image after subtracting the complementary DENSE acquisitions, while contrast-enhancement information was obtained from the T1-weighted magnitude reconstructed image after adding the two DENSE acquisitions. One advantage of the developed technique is that the resulting strain maps are perfectly registered with the Gadolinium (Gd)-enhanced images, which reduces misregistration errors associated with separate acquisition of strain and delayed-enhancement images. The proposed technique may be useful for studying the mechanisms underlying regional myocardial dysfunction in patients with myocardial infarction. It should be noted that other approaches had been previously reported for simultaneously measuring myocardial function and viability information [24,163]. The method in [24] employed magnetization transfer to improve the contrast between normal and Gd-enhanced regions [24], while the method in [163] implemented spin locking for the same purpose.

In a recent development by Le et al [197], the authors developed a single-shot, multi-slice DENSE sequence for simultaneous acquisition of myocardial strain and perfusion information. DENSE images were repeatedly acquired every other heartbeat over 3-4 minutes, covering the first-pass and initial washout of Gadolinium contrast. In the developed technique, the investigators made use of the fact that DENSE images are T1-weighted, and thus myocardium signal intensity is affected by the contrast agent accumulation inside the tissue. The displacement-encoding gradients were combinations of in-plane and through-plane moments. Perfusion and strain information were obtained from the DENSE images magnitude and phase data, respectively. The simultaneous acquisition of myocardial perfusion and strain information allowed for reducing scan time and facilitated perfusion-function information correlation.

### DENSE Image Processing

In addition to the developments made in the DENSE pulse sequence, other developments have been presented for improving DENSE image processing. In [198], Spottiswoode et al presented a method for spatio-temporal phase unwrapping of cine DENSE images, followed by material point tracking and temporal fitting of the trajectories. Motion tracking was improved by fitting curves to the temporal evolution of the trajectories. The improvement in intra-myocardial strain measurements due to the temporal fitting was apparent in strain histograms, and also in identifying regions of myocardial dysfunction in images of patients with myocardial infarction. The developed post-processing technique was reliable, computationally efficient, and relatively user-independent. A couple of years later, Liu et al developed a post-processing method for reconstructing and visualizing dynamic 3-D displacement and strain fields in the LV wall from DENSE images using finite element modeling [199]. The continuous displacement field in the model was mathematically described based on discrete DENSE vectors using a minimization method with smoothness regularization. The developed post-processing method was stable and extended the utility of DENSE, which may provide a patient-specific 3-D model of cardiac mechanics. However, despite of its advantages, finite element modeling suffers from non-specific parameterization and exhaustive computations.

Two studies have recently been published that described methods for employing information about cardiac motion to avoid the use of finite element modeling [200,201]. Gilliam et al developed an automated motion recovery technique termed active trajectory field model (ATFM) for analyzing DENSE images [200]. The developed deformable model exploited both image information and prior knowledge about cardiac motion to automatically recover cardiac motion from a noisy image sequence. The effectiveness of the ATFM method was demonstrated by measuring myocardial motion in 2-D SAX murine DENSE images both before and after myocardial infarction. In the same year, Spottiswoode et al presented a 2-D semi-automated segmentation method that implemented tissue tracking based on the motion encoded into the phase of the cine DENSE images [201]. The total user interaction was reduced to the manual demarcation of myocardium in a single frame. The proposed method had several advantages: segmentation parameters were based on practical physiological limits; contours were calculated for the first few cardiac phases, which have poor blood/myocardium contrast; and the method was independent of the shape of the tissue delineated, which make it applicable to SAX and LAX views.

## SENC

### Original Sequence

**Figure 14:**
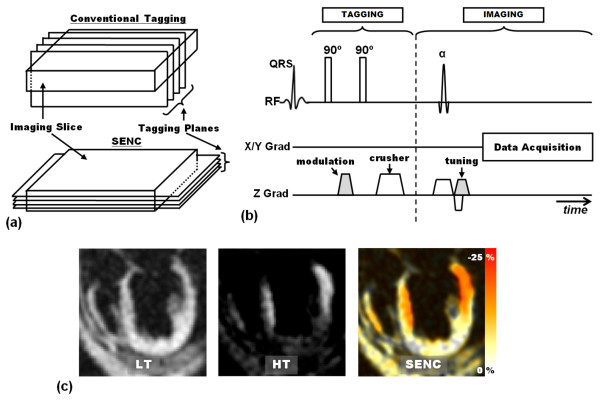
**SENC**. (a) SENC idea. SENC applies tagging as in conventional SPAMM. However, the tag planes are applied parallel to and inside the imaging slice, thus through-plane strain is recorded. (b) SENC pulse sequence. The tagging part is similar to that in conventional SENC, except that the modulating gradient is applied in the through-plane (z-) direction. During the imaging stage, a tuning gradient is applied in the z-direction after slice selection and before data acquisition. By acquiring two images with different tunings, strain information can be decoded. (c) Example of strain images. Low-tuning (LT) image shows non-contracting tissues; high-tuning (HT) image shows contracting myocardium. The LT and HT images are combined to show color-coded strain map. Note that this four-chamber slice shows circumferential strain, and note the big non-contacting apical region.

SENC [26] is a recently-developed CMR technique for measuring myocardial strain (Figure 14). The technique is similar to DENSE in that it is based on a STEAM sequence. However, in contrast to DENSE, strain information in SENC is obtained from the magnitude images. SENC has the advantages of measuring high-resolution strain (on the pixel level) with simple post processing. A color-coded image is obtained that shows through-plane strain map. SENC evolved from the tagging perspective, rather than from STEAM perspective as in DENSE. In contrast to conventional tagging sequences, in SENC tagging is applied in the through-plane direction, which creates a stack of magnetization-saturated planes that lie inside and parallel to the image plane. During the cardiac cycle, these parallel tagged planes move closer together or further apart based on tissue contraction or stretching in the through-plane direction, respectively, which affects the tagging (modulation) frequency. At the imaging time point, different images are acquired with different demodulation (tuning) values. Usually two images are acquired, which are called low-tuning (LT) and high-tuning (HT) images. The LT image is acquired at the same applied tagging frequency to capture signals from static (non-contracting) tissues. On the other hand, the HT image is acquired at a higher frequency (calculated based on slice thickness, applied tagging frequency, and expected strain range) to capture signals from contracting tissues. By combining the information from the LT and HT images, a color-coded strain map is obtained that shows strain values on the pixel level.

Because through-plane strain is measured in SENC, SAX and LAX images show myocardial longitudinal and circumferential strain, respectively. Similar to DENSE, SENC is a black-blood sequence because of the destruction of the tagging pattern in blood during the time in-between tagging application and imaging. SENC has been validated against conventional tagging for measuring both systolic and diastolic LV strain [202], and the results showed that the technique is robust, accurate, and reproducible. The sequence has been used in several research studies [202-207].

### Sequence Developments

Similar to HARP and DENSE, SENC underwent several developments. Fahmy et al developed slice-following SENC (sf-SENC) by combining the slice-following technique with SENC [208]. As in slice-following CSPAMM, a thin slice is tagged (in the z-direction) during the tagging stage, followed by a thicker slice excitation during imaging. sf-SENC allowed for imaging the same myocardial tissue throughout the cardiac cycle.

**Figure 15:**
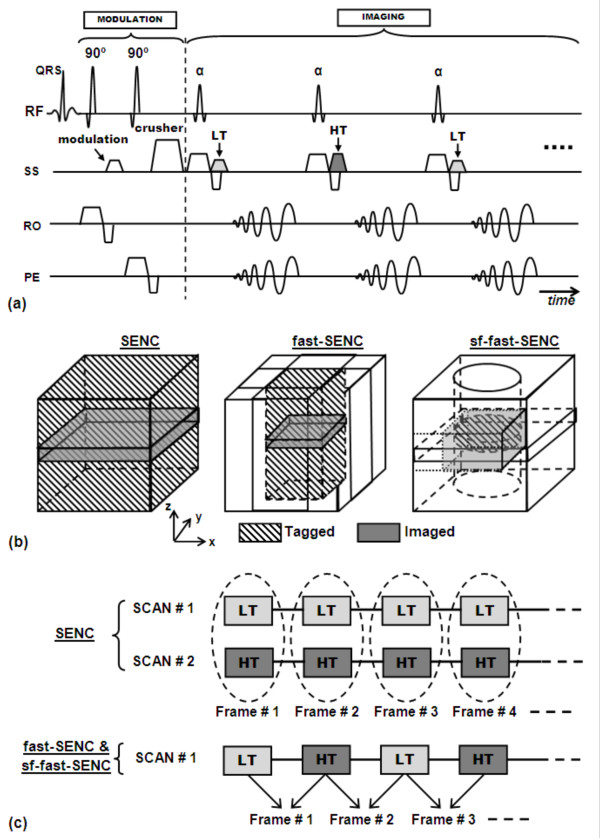
**fast-SENC and sf-fast-SENC**. (a) fast-SENC pulse sequence. The tagging part has two slice-selective RF pulses applied in the x- and y-directions, respectively, to restrict tagging to a small square region, which allows for reducing FOV without much compromising spatial resolution. The second modification in fast-SENC is the implementation of alternating LT and HT tunings in consecutive heart phases to reduce scan time in half. The third modification is the application of spiral acquisition to reduce the imaging time. sf-fast-SENC pulse sequence (not shown) is similar to fast-SENC, except that it replaces the first tagging RF pulse by 2-D cylindrical excitation in the x-y direction and the second RF pulse by a slice-selective pulse in the z-direction to restrict the tagged region to a small disc-shaped region of the intersection of the two excited regions. (b) Shapes showing the tagged and imaged regions in SENC, fast-SENC, and sf-fast-SENC. In SENC, all the body is tagged; thus large FOV imaging is required. In fast-SENC, tagging is restricted to the intersection of the two orthogonal slabs. This allows for using small imaging FOV. In sf-fast-SENC, tagging is restricted to a disc-shaped thin region, which is the intersection of the 2-D cylindrical region and the orthogonal slice. This allows for applying slice following by imaging with a thicker slice that has reduced FOV. (c) In SENC, two scans are required to capture LT and HT images at different heart phases. In fast-SENC and sf-fast-SENC, the tunings are applied alternatively in one scan only.

In another SENC development, Pan et al presented the fast-SENC technique, which is a real-time version of SENC [209] (Figure 15). Three modifications were implemented to allow SENC imaging in a single heart beat (~ 1 second): 1) reduced field of view (FOV) using selective excitation; 2) spiral data acquisition; and 3) interleaved LT and HT tunings. To be able to reduce FOV without introducing aliasing artifacts, the non-selective tagging RF pulses were replaced by slice-selective pulses, one in the x-direction and the other in the y-direction. Thus, the only tagged region is the cuboid resulting from the intersection of the two excited slices (appears as a small square ROI around the heart when looking from the z-direction). This allowed for reducing the acquisition matrix without much affecting spatial resolution. The second modification concerned the use of spiral acquisition, which significantly reduced scan time relative to conventional Cartesian acquisition. Finally, instead of acquiring the series of LT and HT images in two separate scans, they were alternatively acquired in consecutive heart phases during the same scan, which reduced scan time by half. During image processing, each SENC image was used twice (once with the previous image and once with the following image) to construct strain images at the same apparent temporal resolution as if LT and HT images were acquired in separate scans. fast-SENC allowed for real-time strain imaging, which is necessary for certain applications, e.g. during contrast injection, dynamic imaging, or intervention CMR. Korosoglou et al evaluated fast-SENC at 3.0T and showed that the information derived from fast-SENC was equal to that from conventional tagging with prolonged breath-hold [207].

Later, Ibrahim et al combined the features of slice-following and real-time imaging by developing slice-following fast SENC (sf-fast-SENC) [210]. To be able to combine the two features, the tagged region has to be limited in all three directions. It has to have limited expansions in the x- and y-directions to allow for reduced FOV necessary for real-time imaging. The tagged region also has to have limited thickness in the z-direction for applying the slice-following technique. To fulfill these requirements, the first tagging RF pulse was replaced by a 2-D cylindrical excitation pulse in the x-y plane, and the second tagging RF pulse was replaced by a slice-selective pulse in the z-direction. Thus, the only tagged part was the disc-shaped region (that contains the heart) at the intersection of the excited cylinder and orthogonal plane. During the imaging stage, a thick slice (with reduced FOV) was excited to encompass the tagged region despite displacement in the z-direction. Similar to fast-SENC, interleaved tunings and spiral imaging were implemented to reduce scan time to one heart beat.

### C-SENC

**Figure 16:**
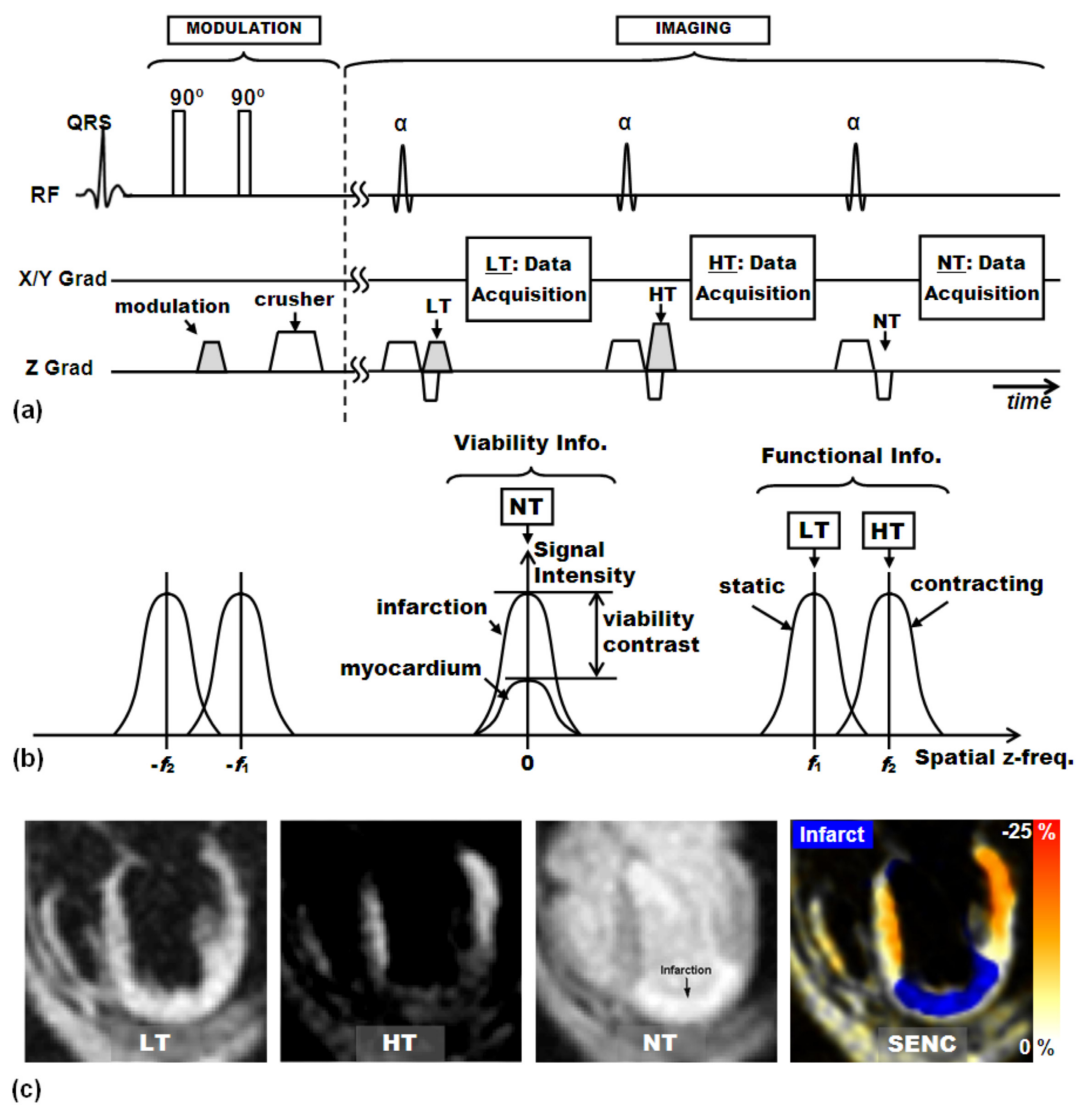
**C-SENC**. (a) C-SENC pulse sequence. The sequence is similar to SENC with the exception that an additional image is acquired without any tuning (no-tuning, NT). (b) Different signals acquired in C-SENC. The LT and HT signals result from non-contracting and contracting tissues, respectively. At zero frequency, another signal peak arises from relaxed magnetization. Infarcted myocardial results in larger signal than normal myocardium 15 minutes after Gd injection due to its preferential accumulation in infarction. (c) Example of C-SENC images. The NT image shows noticeable difference between normal and infarcted myocardium. The C-SENC image is acquired by combining functional information from LT and HT images with viability information from NT image.

Another contribution by Ibrahim et al is composite-SENC (C-SENC) [211], which acquires both myocardial strain and viability information in the same scan (Figure 16). It should be noted that the idea in C-SENC is similar to that developed by Gilson et al for DENSE in [196]. In C-SENC, the SENC sequence was modified to acquire a third image besides LT and HT. This image was acquired without any tunings (thus called no-tuning (NT) image) to capture the non-tagged signal at k-space center. The NT image is T1-weighted because it captures the signal from relaxed magnetization that depends on tissue T1 time constant. The C-SENC sequence is applied 10-15 minutes after Gd injection; thus it shows bright infarction due to shortened T1, secondary to Gd contrast accumulation. In C-SENC, strain information is obtained from the LT and HT images as in conventional SENC, while viability information is obtained from the NT image. When the three images are combined together, a composite image results, which shows both myocardial function and viability information. As the three images are acquired in one scan acquisition, no artifacts are introduced from image misregistration. It should be noted that although myocardium/infarct contrast in the NT image is suboptimal to that in inversion recovery images, the available contrast is sufficient to differentiate between normal and infarcted myocardium after simple intensity histogram analysis.

Another SENC development was presented by Basha et al to improve SENC SNR by combining it with bSSFP imaging [212]. The results showed that the implementation of bSSFP imaging resulted in more accurate results than with spoiled gradient sequences.

### SENC: 3-D Acquisitions

SENC has been combined with other tagging technique to allow for 3-D myocardial motion measurement. In [213], Sampath et al presented a combined SENC and HARP method for measuring myocardial 3-D motion in a single SAX slice. In-plane circumferential and radial strains were calculated from the HARP images, while the through-plane longitudinal strain was calculated from the SENC images. The pulse sequence required only six heartbeats. Two series of HARP images with horizontal and vertical tag lines were acquired in the first and second heartbeats. Two additional series of HARP reference images (at the k-space center) were acquired in the third and fourth heartbeats. Finally, two SENC images with different tunings were acquired in the last two heartbeats. It should be noted that 3-D strain tensor cannot be calculated using the proposed technique as longitudinal shear components are missing. However, multiple SAX slices could be acquired with the proposed technique in a single breath-hold of multiple six-heartbeat length (as many as the number of slices).

In [214], Hess et al combined DENSE and SENC for myocardial 3-D strain measurement in a single slice. The proposed method combined the advantages of DENSE, e.g. high spatial resolution and simple post-processing, with SENC capability of through-plane motion imaging in a single acquisition. In the proposed method, 2-D DNESE was used for in-plane strain measurement in two adjacent slices stacked on top of each other, while SENC was used for through-plane strain measurement in the two slices in order to calculate 3-D strain tensor (the scan required five breath-holds). The use of SENC to measure through-plane motion was faster than imaging two separate slices with DENSE using through-plane encodings. The results demonstrated that the proposed method is capable of reliably quantifying myocardial 3-D strain with high spatial resolution.

## Myocardial Tagging Applications

Myocardial tagging techniques have been used in several clinical and research studies. They have been used for measuring regional LV function [40,41,43,44,71,112,114,122,128,154,203,215-224], RV contractility pattern [117,118,121,142,218,219,225-229], heart rotation and torsion motion [230-240], aging effect on the heart function [123,124,152], coronary artery disease (CAD) [152,241,242], ischemic heart disease [153,205,206,232,243-256], myocardial infarction (MI) [42,67,68,119,125,176,177,204,233,234,247,257-267], dilated cardiomyopathy (DCM) [120,217,237,268-271], hypertrophic cardiomyopathy (HCM) [64,115-118,121,256,272-278], inter-ventricular synchrony [120,203,225,270,272,279-285], valvular diseases [235,236,286,287], congenital heart disease [288-292], post-surgery cardiac function evaluation [281,285,290,293], myocardial elasticity [294-296], and other heart diseases [178,297-302]. In this section, the objectives and main findings in each of these different applications are summarized.

### Regional LV Function

As early as 1991, Clark et al showed the presence of transmural and longitudinal heterogeneity of circumferential shortening in the normal human LV [40]. Subsequent studies confirmed these results, and showed that regional myocardial function is heterogeneous and location dependent in the normal heart [114,215]. In 1995, Naito et all showed that the heterogeneity of myocardial contractility is closely correlated with the myocardial fiber architecture [219]. Two years later, MacGowan et al measured normal fiber shortening in the LV, and the average value was 15% [217]. In 2000, Clarysse et al used CMR tagging to model spatio-temporal myocardial displacement in the normal heart [112]. Later, Rosen et al showed that hypertension and smoking are associated with reduced regional LV function in asymptomatic individuals [301]. Other investigators used 3-D tagging for studying LV regional function [43,122,128]. Advanced tagging techniques have been also used for generating LV myocardial strain maps [154,203,223]. In 2006, Rodriguez et al were able to calculate tissue volume change with 1% precision using myocardial tagging [224]. Subsequently, Cupps et al tested the capability of multiparametric strain analysis for optimizing the quantification of regional and transmural myocardial contractile function [222]. Recently, Nasiraei-Moghaddam et al used CMR tagging to show the presence of a functional helical myocardial band in LV [302].

LV function has been also studied in the presence of RV pressure overload using CMR tagging [117,118,225]. In [117], the investigators studied the relationship between the distortion of the LV circular shape and regional LV function in the presence of RV pressure overload, and they concluded that in this group of patients, the septal function is reduced with nonuniform distribution in endocardial circumferential shortening. In [118], the investigators reported that the altered distribution of regional circumferential shortening in patients with RV pressure overload results in an increased heterogeneity of regional LV systolic motion. Similar results were presented in [225].

The aging effect on myocardial LV function has been studied using CMR tagging. In 2003, Fonseca et al showed that patterns of regional nonuniformity of myocardial relaxation are altered in a consistent fashion with aging [123]. In the same year, Oxenham et al found significant prolongation and reduction of myocardial relaxation in older compared with young normal individuals [124]. Similar finding was reported by Castillo et al using the HARP technique, and the investigators reported that HARP provides high inter- and intra-observer agreements in the results [152].

### Regional RV Function

Despite of the complex shape and thin wall of the RV that make it challenging to study its regional function, RV was the subject of many CMR studies in order to better understand how it works. RV function is compromised in many cardiovascular diseases that affect the pulmonary circulation. Furthermore, RV dysfunction may affect the LV normal operation due to the interventricular dependence, which makes studying RV mechanics as important as that of the LV. In 1995, Naito et al conducted a study to quantitate the contraction of the RV free wall and to identify normal performance compared with the LV [219]. One year later, Young et al used CMR tagging to reconstruct RV 3-D motion [142]. A key study then came out in 1998 by Fayad et al, in which the investigators compared the regional variation of RV free wall and interventricular septum in normal subjects and in patients with pulmonary hypertension [227]. Another study was presented two years later that analyzed RV motion in normal volunteers and patients with RV hypertrophy, and the results showed a noticeable difference between the two measurements [121]. Recently, two studies were conducted, in which RV regional function was assessed using SENC [228,229].

### Torsion Motion of the Heart

Although strain and strain rate are the mostly studied variables using CMR tagging, several studies investigated the wringing (torsion) motion of the heart. In 1992, Azhari et al studied myocardial transmural twist using CMR tagging [240]. Later, Dong et al showed that there is a potential for the torsion-volume relation to provide a load-independent measure of myocardial contractility [230]. Another important study came out in 2000, in which Lorenz et al studied normal human LV twist throughout systole using CMR tagging [238]. Dong et al conducted another study, in which they showed that the rate of recoil of myocardial torsion provides an assessment of LV relaxation [231]. Other studied have been conducted to study the effect of different cardiovascular diseases on myocardial torsion motion, as will be explained later.

### CAD and Ischemic Heart Disease

Out of the importance of studying CAD for better understanding of the disease pathophysiology, the Multi-Ethnic Study of Atherosclerosis (MESA) was initiated in 2000 to examine the predominance and advancement of subclinical cardiovascular disease. Using CMR tagging, the investigators studied the relationship between regional heart function and atherosclerosis risk factors, and important results were presented based on this study. For example, Edvardsen et al investigate the relationship between regional LV systolic function and coronary calcium score [241]. The results showed that regional LV measures serve as subclinical surrogates for CAD. In the same year, Fernandes et al showed that greater carotid intima-media thickness is associated with reduced systolic and diastolic myocardial function [242].

Several studies have been conducted that used CMR tagging to study myocardial function in the presence of ischemia. As early as 1989, Prinzen et al used CMR tagging to investigate the relationship between myocardial blood flow and fiber shortening in the ischemic border zone [255]. The investigators found major discrepancies between perfusion and deformation in the periphery zone of ischemia. Later, Kroeker et al found that ischemia has profound effects on the dynamics of apex rotation [232]. The results were later confirmed by Garot et al, who showed that acute myocardial ischemia is responsible for a decrease in LV twist that is related to global LV function [233]. In 1998, three articles were published that described the importance of combining tagging with perfusion for evaluating ischemic heart disease [246,247,252]. In [252], Kraitchman et al showed the importance of integrated CMR assessment of regional function and perfusion in canine MI. In [247], the authors showed that the response of intramyocardial function to low-dose dobutamine after reperfused MI can be quantified with CMR tagging. In [246], the investigators found that combining tagging with dobutamine stress helps detecting recovery of hibernating myocardium after revascularization. In 1999, Scott et al used CMR tagging to study the effect of dobutamine on regional LV function [253]. In the same year, Rogers et al showed that early contrast-enhanced CMR predicts late functional recovery after reperfused MI [244]. In a similar study, Gerber et al reported that the lack of delayed hyperenhancement has better diagnostic accuracy in predicting functional improvement in dysfunctional segments [245]. An important study came out in 2003, in which Kuijpers et al showed that stress CMR with myocardial tagging could reliably detect confirmed new wall motion abnormalities than stress CMR alone [248]. In the same year, Kraitchman et al used HARP for detecting the onset of ischemia during stress imaging [153]. Later, Samady et al showed that electromechanical mapping parameters can identify segments that improve rest function and retain contractile reserve early after revascularization [250]. Furthermore, Paetsch et al showed that the use of myocardial tagging may reduce the need for high-dose dobutamine stress in stress perfusion scans [251]. A similar study showed that the response of systolic strain to low-dose dobutamine has significant promise in discriminating between viable and nonviable myocardium [249]. Another study showed that lower myocardial flow reserve is related to reduced regional function in asymptomatic individuals [301]. Recently, two studies were conducted, in which SENC has been used for detecting inducible ischemia during dobutamine stress scans [205,206].

### Myocardial Infarction

In 2001, Gotte et al showed that strain analysis is more accurate than wall thickness analysis in discriminating dysfunctional from functional myocardium [261]. In [259], the investigators showed that LV dilatation and eccentric hypertrophy during remodeling are associated with persistent differences in segmental function between adjacent and remote noninfarcted regions. In 1995, Lima et al found that nonreperfused transmural myocardial infarction is characterized by marked reduction and reorientation of principal strains [257]. In the same year, Chen et al used SPAMM to detect regional MI [42]. More detailed information about myocardial kinematics after MI was presented by Kramer et al, who showed that patients after first anterior MI with single-vessel disease demonstrate reduced intramyocardial circumferential shortening throughout the LV [262]. An important study then came out in 1997, where the investigators showed that early after MI, regions with dysfunction, normal function, and hyperfunction can be delineated with CMR tagging [258]. Two years later, two studies were published that showed the capability of strain mapping of characterizing ischemic injury [119,263]. Regional myocardial function has also been studied in the regions around the infarcted zone [260,264,266,267]. In 2000, Gerber et al showed that in the early healing phase of acute MI, the extent of microvascular obstruction in infarcted tissue relates to reduced local myocardial deformation and dysfunction of noninfarcted adjacent myocardium [260]. Similarly, Kramer et al reported that there are more diffuse abnormalities in regional mechanical function than simply within the zone of healed infarction [264]. Recently, the investigators implemented CMR tagging to determine the mechanical behavior of LV myocardium in the peri-infarct zone [267]. Studies have been also conducted to investigate the difference between regional myocardial function in healthy and infarcted rodents [67,68]. Advanced tagging techniques have been also used to examine regional myocardial function in the presence of MI [68,176,177,202,204]. In [176], DENSE was used to study the anatomical correlation between abnormal myocardial electromechanics and infarct geometry. In [177], DENSE was used to determine whether edema imaging could delineate the area at risk in reperfused MI. In [204], SENC was implemented to discriminate between different transmurality states in patients with acute MI. CMR tagging has been also used to investigate the effect of passive constraints on akinetic area development secondary to acute MI [293].

### Dilated Cardiomyopathy

In 1997, MacGowan et al showed that normal fiber shortening is 15%, and this value is markedly reduced in DCM [217]. Two studies came out in 2000 to study mechanical dyssynchrony in DCM [120,270]. Later, Young et al showed a consistent pattern of regional heterogeneity of myocardial strain in DCM [268]. Recently, two studies examined systolic torsion pattern in DCM using CMR tagging [237,239].

### Hypertrophic Cardiomyopathy

In 1995, Beache et al showed that strain rate characterization provides an objective measure of disease course in HCM [276]. In 1992, Maier et al observed a reduced cardiac rotation in patients with HCM, mainly in the posterior region [275]. Similar results were reported in [115,277]. In 1994, Dong et al reported that the myocardium in patients with HCM is heterogeneously thickened compared to healthy subjects [116]. Later, Mishiro et al found that systolic LV wall asynchrony occurs in patients with HCM [272]. In 2000, Bergey et al used CMR tagging to correctly diagnose focal HCM simulating a cardiac mass [278]. Later, two studies confirmed the impaired diastolic function in HCM [64,274]. Recently, Kim et al showed that impaired circumferential shortening occurs in delayed hyperenhancement areas in HCM [273].

### Interventricular Synchrony

In 1998, McVeigh et al used CMR tagging to image asynchronous mechanical activation of the paced heart [283]. In a related work, Wyman et al studied the effect of pacing site on mechanical activation pattern using myocardial tagging [281]. In 2000, Pusca et al assessed the effect of synchronized direct mechanical ventricular actuation on LV dysfunction [285]. Zwanenburg et al conducted two interesting studies to map the timing of cardiac contraction with high temporal resolution CMR tagging [279], and to study the relationship between peak time and onset time of myocardial shortening [280]. In 2005, Helm et al analyzed cardiac dyssynchrony using circumferential versus longitudinal strain [282]. In the same year, Lardo et al presented an important review article about the use of CMR for assessment of ventricular dyssynchrony [284]. Recently, SENC has been used for assessing regional strain and timing of contraction of the LV free wall in healthy individuals [203].

### Valvular Heart Disease

In 1993, Villari et al used CMR tagging to evaluate LV structure-function interplay in aortic valve disease, and concluded changes in collagen architecture are associated with altered systolic function and passive diastolic properties in aortic valve disease [287]. Later, Ungacta et al quantified the differences in regional LV systolic deformation before and after surgery for aortic insufficiency [286]. In 1999, Stuber et al studied the alterations in cardiac torsion and diastolic relaxation of the LV in patients with aortic stenosis, and concluded that delayed diastolic untwisting in the pressure-overloaded hearts may lead to diastolic dysfunction [235]. In a similar work, Nagel et al evaluated the time course of rotational motion of the LV in patients with aortic valve stenosis [234]. In another work, changes in LV systolic rotation and contraction in aortic stenosis were examined before and after surgical valve replace [236].

### Congenital Heart Disease

Fogel et al presented a series of studies that included the implementation of CMR tagging for evaluation of different congenital heart diseases [288,289,291,292]. In [288], the investigators compared the function of single right ventricles with systemic right ventricles in a dual-chamber circulation. In the same year, the changes in systolic regional wall deformation was studied in patients with single ventricle after surgical intervention [292]. In [291], the authors used CMR tagging to study the biomechanics of the deconditioned LV. In [289], Fogel et al continued their efforts to study the effect of the RV on systemic LV mechanics. Later, Donofrio et al used CMR tagging to measure regional wall motion and strain of transplanted hearts in pediatric patients [290].

### Myocardial Elasticity

Some studies have been conducted to measure myocardial elasticity with CMR tagging. In 2003, SENC was implemented for detecting stiff masses [294], and the results indicated that SENC could be used for assessing myocardial elasticity. A couple of years later, Wen et al developed a CMR-based technique that incorporated DENSE and phase contrast velocity mapping to assess regional stress/strain gradients in the LV myocardium during diastole [295]. The results were used to calculate myocardial regional elastic moduli and viscous delay time constants. Recently, Robert et al presented a method based on DENSE for measuring myocardial viscoelastic parameters with the help of an electrodynamic transducer [296]. The proposed technique was validated in a phantom, excised pig heart specimen, and in vivo experiments.

### Other Heart Diseases

In addition to the studies mentioned above, CMR tagging has been also used for evaluating the heart condition in other cardiovascular diseases. In 1999, Kojima et al investigated the diagnosis of constrictive pericarditis by CMR tagging [297]. In a related work, Spottiswoode et al assessed postoperative systolic septal wall motion abnormality following pericardiectomy [193]. In 2000, Blom et al showed that dynamic cardiomyoplasty decreases myocardial workload [298]. Kramer et al studied reverse remodeling and improved regional function after repair of LV aneurysm [299]. Later, Setser et al studied the effects of partial left ventriculectomy on myocardial mechanics [269]. In 2005, Ashford et al studied the occult cardiac contractile dysfunction in dystrophin-deficient children [271]. Recently, Fernandes et al found that arterial stiffness is associated with regional ventricular systolic and diastolic dysfunction [300].

## Summary and Future Directions

This review presented a plethora of ideas and techniques that have been used for developing different CMR tagging techniques. Although the magnetization saturation technique opened the door for quantifying myocardial deformation in 1988, the technique showed not to be clinically applicable due to the sequence inefficiency for creating the tag lines. SPAMM addressed this limitation, and it remains to be the gold-standard tagging technique up to the current day. CSPAMM is also widely used because of its high SNR and sharp tags. However, because it requires double the scan time as SPAMM, CSPAMM may not be appropriate for patients who cannot hold their breath for a long time. Nevertheless, it can be combined with navigator echo for free-breathing imaging. Unfortunately, some magnetic resonance imaging systems do not even have the CSPAMM sequence on their commercial cardiac package. DANTE is not widely used as SPAMM or CSPAMM.

This article showed that some sequence developments were made to address specific shortcomings in tagging, e.g. tagging contrast fading during the cardiac cycle or slice through-plane displacement. Other developments involved combining tagging with different data acquisition techniques, e.g. EPI or bSSFP, mainly to reduce scan time. Nonetheless, the exhaustive post-processing needed to extract and track the tag lines remains the main obstacle of basic tagging techniques (SPAMM, CSPAMM, and DANTE). Among the different processing techniques, active contour methods showed accurate results and efficient processing. Nevertheless, the real post-processing challenge occurs when analyzing a 3-D set of cine tagged images, which requires significant processing time.

Advanced tagging techniques have the advantage of simple post-processing. However, they are more sophisticated and less straightforward than the basic techniques. Similar to the basic tagging techniques, the advanced techniques underwent different developments to improve image quality (enhance SNR, reduce scan time, remove artifacts) or to extend into 3-D capability. Because different techniques have different advantages, some efforts have been made to merge some of these techniques together to combine their advantages.

It is interesting to see the similarities between different advanced tagging techniques, and to notice that they followed similar paths of developments. For example, although the SENC technique was described in terms of tagging (in the z-direction), it can be seen also as a STEAM pulse sequence consisting of the three STEAM stages: modulation, mixing, and demodulation. From this perspective, the SENC sequence is basically equal to DENSE with displacement encoding in the z-direction. Conversely, DENSE can be interpreted in terms of tagging, where the tag planes are applied in the desired direction of motion, and then the spectral peak is restored at k-space center before data acquisition. However, the difference between the two techniques is that DENSE decodes motion information from the phase data, while SENC depends on acquiring more than one image with different modulations (in the same direction) and extracting the motion information from the magnitude images. By the same line of thought, the relationship between HARP and DENSE can be explained. Both HARP and DENSE deal with the phase of the modulated images. However, HARP extracts the spectral peak of the tagged image using bandpass filter to create the HARP image, which looks very similar to the originally tagged image (shows parallel stripes at the position of taglines). On the other hand, DENSE restores the tagging (modulation) spectral peak at the k-space origin at the demodulation stage before data acquisition, and deals with the resulting phase information. Thus, the resulting DENSE magnitude image does not have the striped pattern shown in HARP.

The question to raise now is what next? What areas of improvements are most needed in CMR tagging, and what directions will outline the future of the technique? One imperative requirement is improving SNR. Advanced tagging techniques, e.g. HARP, DENSE, and SENC, share the limitation of low SNR. All of them have 50% cut in SNR due to the pulse sequence design or processing nature. Therefore, current real-time images have low spatial resolution, and high spatial resolution images suffer from low SNR. Thus, enhancing SNR will lead to better quality images and more accurate results. The current trend towards high-field (3.0T and above) clinical scanners will help improve SNR.

Another interesting development would be to have strain maps directly displayed on the scanner console on-the-fly. Unfortunately, current data processing techniques are conducted offline, even though some of them are simple and fast. The ability to provide online results will allow the operator to adjust the imaging parameters on the fly or switch between sequences during the CMR exam, which will allow for optimized imaging for each patient. This development needs the scanner software to be programmed to process the raw data and display the end results online.

One more trend for tagging improvement is modifying the pulse sequences to allow for acquiring composite information in the same scan. Some examples have been presented about combining viability and function information in DENSE and SENC. The major advantage of such techniques, besides reducing scan time, is alleviating misregistration problems, which allows for more accurate correlation between different measurements. More research is needed in this direction to help more understand different heart diseases.

Finally, more large studies that include CMR tagging should be conducted to create a database of the ranges of different regional myocardial measurements in different heart diseases. This database would act as a reference, against which patient results could be compared for quick assessment of the patient condition.

## Competing interests

The author declares that they have no competing interests.

## Author's information

The author received his Master and PhD degrees from Johns Hopkins University on medical imaging. He currently works as an Assistant Professor of Radiology in University of Florida, USA. The author has numerous publications in the field of CMR, and provides scientific revision for IEEE, MRM, JCMR, and ISMRM in this field.
